# Exosome prospects in the diagnosis and treatment of non-alcoholic fatty liver disease

**DOI:** 10.3389/fmed.2024.1420281

**Published:** 2024-07-31

**Authors:** Amirhossein Tamimi, Mona Javid, Nasrin Sedighi-Pirsaraei, Arian Mirdamadi

**Affiliations:** School of Medicine, Guilan University of Medical Sciences, Rasht, Iran

**Keywords:** NASH, non-alcoholic fatty liver, exosome, NAFLD, extracellular vesicle (EV), microRNA

## Abstract

The growing prevalence of NAFLD and its global health burden have provoked considerable research on possible diagnostic and therapeutic options for NAFLD. Although various pathophysiological mechanisms and genetic factors have been identified to be associated with NAFLD, its treatment remains challenging. In recent years, exosomes have attracted widespread attention for their role in metabolic dysfunctions and their efficacy as pathological biomarkers. Exosomes have also shown tremendous potential in treating a variety of disorders. With increasing evidence supporting the significant role of exosomes in NAFLD pathogenesis, their theragnostic potential has become a point of interest in NAFLD. Expectedly, exosome-based treatment strategies have shown promise in the prevention and amelioration of NAFLD in preclinical studies. However, there are still serious challenges in preparing, standardizing, and applying exosome-based therapies as a routine clinical option that should be overcome. Due to the great potential of this novel theragnostic agent in NAFLD, further investigations on their safety, clinical efficacy, and application standardization are highly recommended.

## Highlights

- Different exosomes participate in the development of non-alcoholic fatty liver disease.
- Exosomes are ideal biomarkers for the diagnosis of non-alcoholic fatty liver disease.
- Exosomal microRNAs can differentiate the different stages of non-alcoholic fatty liver disease.
- Stem cell-derived exosomes can ameliorate the course of non-alcoholic fatty liver disease.
- Exosomal blocking can prevent non-alcoholic fatty liver disease progression.

## 1 Introduction

Affecting one out of four adults globally, Non-alcoholic fatty liver disease (NAFLD) remains a growing worldwide health issue (1). It is highly associated with metabolic syndrome and commonly accompanies insulin resistance, obesity, and dyslipidemia (1). Moreover, it is increasing due to the Growing obesity and diabetes prevalence (2, 3).

NAFLD is a histological definition describing macrovesicular steatosis in more than 5% of hepatocytes in people with no or little alcohol consumption. It includes two major types: non-alcoholic fatty liver (NAFL) and non-alcoholic steatohepatitis (NASH). Non-alcoholic fatty liver (NAFL), or simple steatosis, is a form of NAFLD. While NAFL is typically accompanied by a lower risk of liver-related mortality, it can progress to non-alcoholic steatohepatitis (NASH) (4). NASH is more commonly associated with cirrhosis and hepatocellular carcinoma (HCC), both of which can lead to liver-associated death (4). Histologically, NASH consists of ballooning degeneration and lobular inflammation along with steatosis, with or without perisinusoidal fibrosis (5). However, present theories propose a dynamic two-way cycling between NAFL and NASH, with fibrosis advancing slowly in most patients (4). The reality that the initiation of progressive fibrosis ultimately determines clinical results raises doubts about the importance of differentiating between NAFL and NASH (4).

Although there has been a lot of effort in developing non-invasive tools for NAFLD diagnosis, the diagnosis of NAFLD is mainly based on liver biopsy (6, 7). However, it is an invasive and costly procedure, and, considering the prevalence of the disease, impractical. Therefore, non-invasive diagnostic methods that could be accurate can be crucial in this area. Along with lifestyle modification and weight loss, several agents, including pioglitazone, semaglutide, and obeticholic acid, have shown promise in the therapy of NAFLD (7). However, with more than 50 treatment agents currently being studied in different clinical phases, the treatment of NAFLD remains challenging (7).

Exosomes are nanosized extracellular vesicles that have significant roles in the pathogenesis of different diseases (8). Recently, their theragnostic potentials have been broadly discussed in various diseases, including cardiac, renal, hepatic, and neurological diseases, as well as neoplasms (8). In the presenting paper, we aimed to review the potential and perspectives of exosomes as a diagnostic and therapeutic agent in NAFLD.

## 2 Exosomes

Exosomes are bi-lipid membrane extracellular vesicles sized from 50 to 140 nm (9–11). They are secreted by nearly all cells and can be found in all body fluids (12–15). Exosomes carry a wide variety of proteins, DNAs, RNAs, and lipids, with specific characterization based on the secreting cell (16–19). They have higher levels of specific proteins [such as heat shock protein 70 (HSP 70), tetraspanins (CD9, CD 63, CD 81, and CD 82), ALG-2-interacting protein X (ALIX), tumor susceptibility gene 101 (TSG101)] and lipids [such as flotillin and glycosylphosphatidylinositol-anchored protein (LBPA)] (20–22).

Exosome biogenesis involves complex intracellular pathways with distinct mechanisms for different EV subtypes (23). The endosomal system plays a central role in exosome biogenesis (24). Traditionally, the endosomal sorting complex required for transport (ESCRT) machinery has been considered essential for the biogenesis of exosomes (24). The biogenesis of exosomes involves four key steps: cargo sorting, the development and maturation of MVBs, the transportation of these MVBs, and finally, the fusion of MVBs with the cellular membrane (25). Each process is modulated through the competition or coordination of multiple mechanisms, whereby diverse repertoires of molecular cargos are sorted into distinct subpopulations of exosomes, resulting in the high heterogeneity of exosomes.

Recognition of Ubiquitinated Cargo is the first step in exosome biogenesis (24). The process begins with the recognition of ubiquitinated cargo by the ESCRT-0 complex (24). Ubiquitination is a post-translational modification where a small protein called ubiquitin is attached to a target protein (26). This serves as a recognition signal for ESCRT-dependent cargo sorting. Once the ubiquitinated cargo is recognized, it is sequestered into endosomal microdomains by the ESCRT-I and ESCRT-II complexes (25). These complexes also initiate the budding of the endosomal membrane into the lumen of the endosome, forming the nascent ILV. The ESCRT-III complex is responsible for driving the budding process to completion, leading to the formation of ILVs (24). It does this by assembling into a spiral-shaped structure on the endosomal membrane, which constricts the neck of the budding ILV. ILVs are formed within the lumen of an endosome through the inward budding of the endosomal membrane. When multiple ILVs accumulate within an endosome, it is then referred to as a multivesicular body (MVB). MVBs can either fuse with lysosomes for degradation or with the plasma membrane, releasing ILVs as exosomes.

Recent findings suggest the existence of an alternative pathway for sorting exosomal cargo into MVBs in an ESCRT-independent manner (24). Nonetheless, the pathways may not be completely distinct (27). This ESCRT-independent pathway seems to depend on raft-based microdomains for the lateral segregation of cargo within the endosomal membrane (28). For instance, the nSMase2-ceramide pathway is vital for ESCRT-independent exosome biogenesis (29). Besides, Tetraspanins are a family of proteins that play a crucial role in the biogenesis of exosomes, including the ESCRT-independent pathway (30). They are characterized by their four transmembrane domains, which allow them to interact with various other proteins, cholesterol, and gangliosides. These interactions lead to the formation of tetraspanin-enriched microdomains (TEMs) on the membrane (30). These TEMs can influence membrane bending and actin polymerization, which are essential steps in the formation of multivesicular bodies (MVBs) and, subsequently, exosomes (30). In addition to their role in exosome biogenesis, tetraspanins are also sorted into exosomes in an ALIX- and ESCRT-III-dependent manner (31). This sorting process occurs independently of other ESCRTs but requires lysobisphosphatidic acid (LBPA) *in vivo*.

Lately, exosomes have got much attention in diagnostics. They constitute a key intracellular communication system and act as a key factor in the pathogenesis of different diseases via intercellular signaling cascades (23, 32–35). Therefore, detecting exosomes, molecules carried by them such as nucleic acids, and their surface proteins may be used as an early, non-invasive, and potentially accurate diagnostic tool in different diseases (8). Their potential diagnostic value has been proven in cancers and diseases of the lungs, kidneys, liver, and central nervous system (36–42).

Exosome offers favorable features as a therapeutic agent. It has the same homing behavior as the secreting parent cell (43). Additionally, it benefits from a bilipid membrane, which allows it to carry considerable amounts of contents and protect its cargo from degradation by chemicals and enzymes. Considering that along with their targeted activity, exosomes are fruitful options in drug delivery applications. Moreover, they have significantly lower immunogenicity than virus-based drug delivery systems and liposomes (44). Besides, exosomes derived from mesenchymal stem cells provide immunomodulatory and anti-inflammatory effects, promote cellular viability, and facilitate cellular proliferation and neoangiogenesis (45–50). In certain settings, exosomes can have regenerative and homeostatic, thus therapeutic effects on diseased tissue (51). On top of that, exosomes lack risks of carcinogenesis and immune response to cancers and infections, compared with cell therapies, offering high therapeutic potential with appreciable safety (9).

## 3 Pathophysiology of NAFLD

The complex pathophysiology of NAFLD has not yet been fully clarified. However, it is believed that it is related to the interaction among genetic, environmental, and individual factors (52).

There are different hypotheses about NAFLD pathophysiology. In the past, the “two-hit hypothesis” was used to explain the mechanism of NAFLD and NASH pathophysiology, but currently, the “multiple-hit model” is suggested (52). According to the two-hit model, the initial phase, often referred to as the “first hit,” involves the build-up of fat, specifically triglycerides, within the liver tissue and the development of insulin resistance. These elements are considered the key drivers of hepatic steatosis when the accumulation surpasses the threshold of 5% (53). The “second hit” is characterized by alterations in the levels of inflammatory cytokines and adipokines, mitochondrial dysfunction, and oxidative stress. These changes can trigger necroinflammation and fibrosis within the liver tissue (54, 55). However, recent investigations have shown that the “two-hit hypothesis” cannot fully explain the exact mechanism of human NAFLD. Therefore, the “multiple-hit model” is currently the most recognized theory. It suggests a broader metabolic dysfunction due to the interplay of genetic and environmental influences, as well as alterations in the crosstalk between the liver and various organs and tissues, such as the adipose tissue, pancreas, and gut (52, 54–57). Despite this, the initial stages, or “first hits,” are still believed to be the accumulation of fat in the liver triggered by obesity and insulin resistance (52).

NASH and NAFLD begin with the excessive accumulation of triglycerides (TG) in the liver (58). The fatty acids used for this hepatic TG accumulation are derived from three sources: (I) dietary fat taken up in the intestine, (II) *de novo* lipogenesis from glucose and fructose, and (III) dysregulated adipocytes' triglyceride lipolysis leading to excessive fatty acid transport to the liver (58–60). When the liver's capacity to utilize carbohydrates and fatty acids, as the primary metabolic energy substrates, is impaired, toxic lipid species are accumulated in hepatocytes. It, in turn, induces hepatocellular injury and death, which results in genomic instability and fibrogenesis that put the liver at risk of cirrhosis and hepatocellular carcinoma. However, the specific toxic lipid species that promote cell injury are not fully recognized (6). Lipotoxic lipids accumulation causes hepatocellular injury through endoplasmic reticulum (ER) stress (61), a dysregulated unfolded protein response (UPR) (62), activation of the inflammasome and apoptotic pathways (63), an increased hepatic Hedgehog (Hh) signaling, and inflammation (64). There are many external factors contributing to hepatocellular injury, including insulin resistance (65), adipokine dysregulation (66), hepatic ATP depletion (67), uric acid-induced hepatocyte mitochondrial dysfunction (68), and the effects of intestinal microbiota products (69). Fibrogenesis results from extracellular signaling from injured hepatocytes, liver sinusoidal endothelial cells, activated Kupffer cells, T cells, B cells, and natural killer cells, promoting hepatic stellate cells (HSCs) activation. Activated HSCs transdifferentiate into fibrogenic myofibroblasts that can produce extracellular matrix proteins at a higher rate than their degradation (70). Finally, Progressive fibrosis leads to cirrhosis, which subsequently promotes portal hypertension and liver failure, which is the main reason for liver-related mortality in NAFLD (6).

## 4 Exosome in NAFLD

### 4.1 Role of exosomes in NAFLD pathophysiology

#### 4.1.1 Exosomes in the pathogenesis of NAFLD

**Figure 1:**
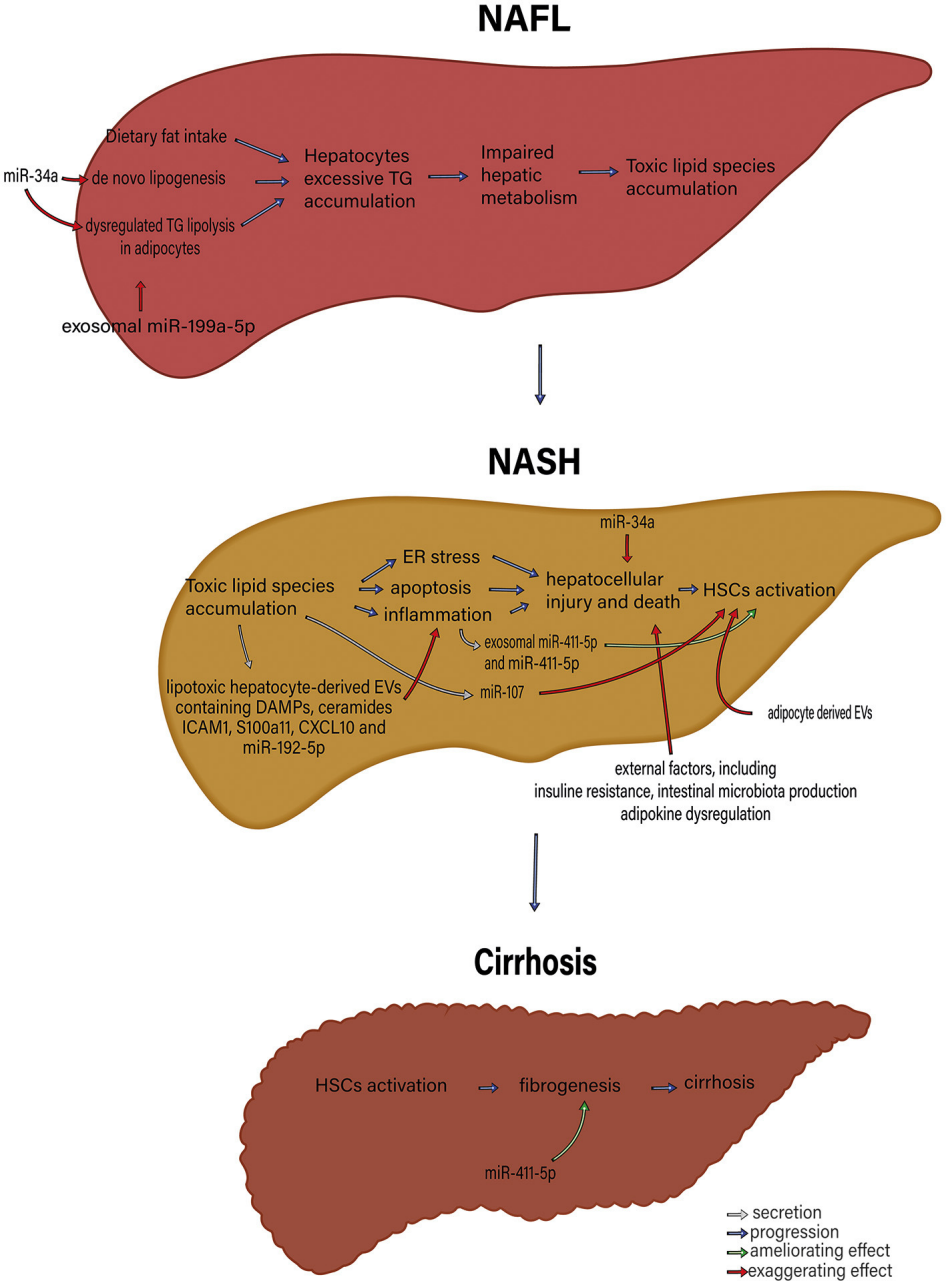
The roles of exosome and exosomal miRNAs in NAFLD pathophysiology. microRNAs, including miR-34a and exosomal miR-199a-5p, that control *de novo* lipogenesis and dysregulated TG lipolysis in adipocytes significantly induce excessive hepatocyte TG accumulation. Subsequently, TG accumulation in hepatocytes gives rise to impaired hepatic metabolism and toxic lipid species accumulation. The accumulation of toxic lipid species leads to ER stress, apoptosis, and inflammation. These changes contribute to hepatocellular injury and death, followed by HSC activation. MiR-34a, exosomal miR-411-5p and miR-411-5p, miR-17, adipocyte-derived EV, lipotoxic hepatocyte-derived EVs containing DAMPs, ceramides, ICAM1, S100a11, CXCL10 and miR-195-5p and external factors including insulin resistance, intestinal microbiota production, and adipokine dysregulation participate in various parts of this process. HSC activation results in fibrosis and, eventually, cirrhosis. miR, microRNA; TG, triglyceride; ER, endoplasmic reticulum; DAMPs, Damage-associated molecular patterns; ICAM-1, Intercellular Adhesion Molecule 1; HSCs, Hepatic stellate cells; EVs, extracellular vesicles.

**Figure 2:**
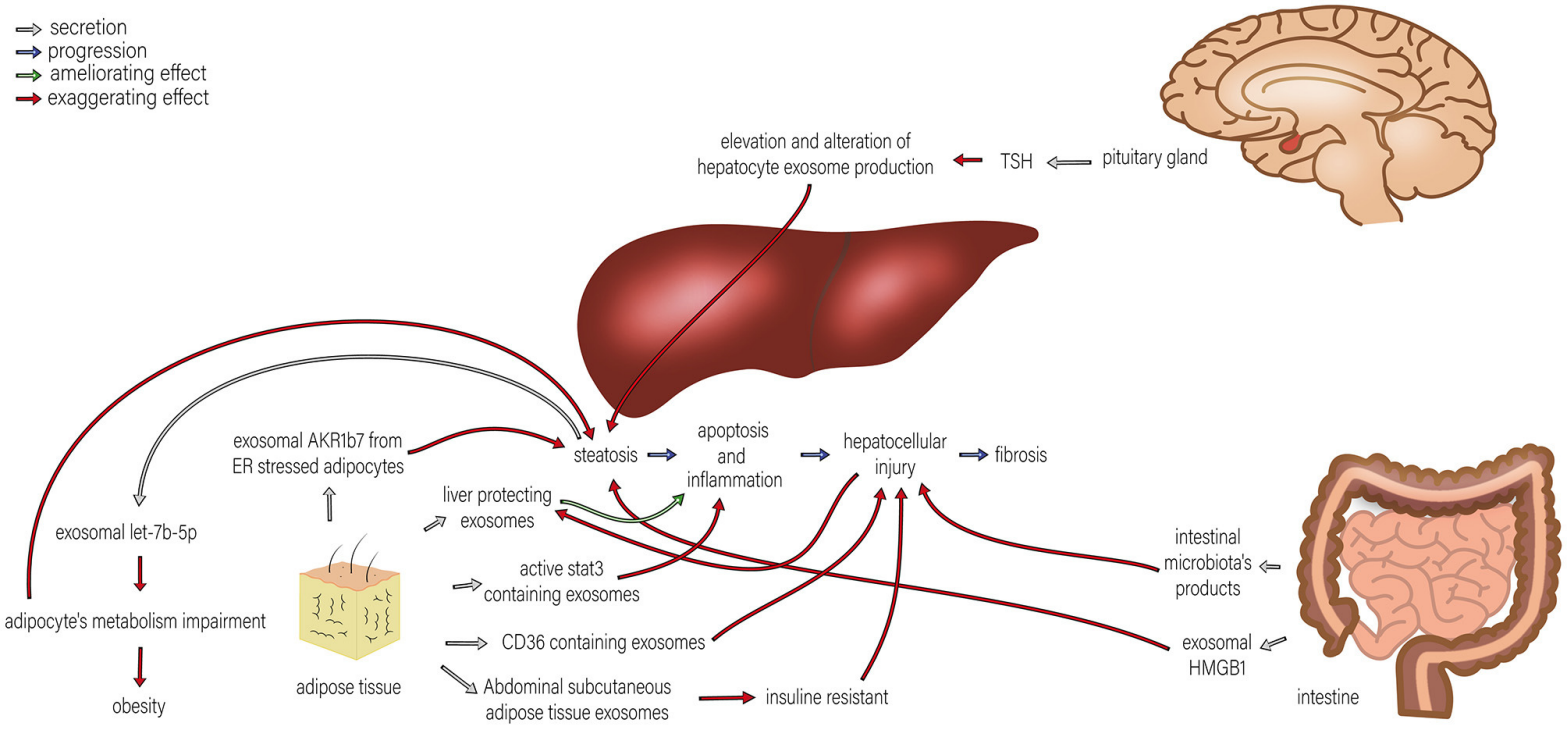
The roles of exosomes in the interactions of the liver and pituitary gland, intestine, and adipose tissue in NAFLD. Different organs are involved in the development of NAFLD, and their interactions with the liver play significant roles in NAFLD progression. By TSH regulation, the pituitary gland alters hepatocyte exosome production, which exaggerates steatosis. TSH stimulation of HepG2 cells leads to exosome release and upregulation of proteins involved in steatosis. Adipose tissue induces progression in various steps of NAFLD, including steatosis, apoptosis, inflammation, and hepatocellular injury by exosomal AKR1B7 released from ER stressed adipocytes, active stat3 containing exosome, CD36 containing exosomes and insulin resistance caused by abdominal subcutaneous adipose tissue exosomes. Therefore, adipocytes' metabolism impairment promotes steatosis. Moreover, steatosis can also exaggerate the metabolism impairment of adipocytes through exosomal let-7b-5p. On the other hand, liver-protecting exosomes originating from adipose tissue can ameliorate apoptosis and inflammation. The intestine can also affect NAFLD development in two ways: (1) intestinal microbiota products can amplify hepatocellular injury, and (2) exosomal HMGB1 increases steatosis.

Several recent studies point to a significant role of Extracellular Vesicles (EVs) in the pathophysiology and progression of NAFLD and NASH. EVs play a central role in normal intercellular communication. Different types of liver cells, including human adult liver stem cells, cholangiocytes, hepatocytes, hepatic dendritic cells, and hepatic stellate cells, function as both exosome-secreting and exosome-targeted (42). Various liver conditions, including NAFLD, seem to increase the basal EV's secretion (71). Povero et al. (72) have shown a considerable rise in the concentration of EVs in the liver and blood of diet-induced NAFLD animals. Normal hepatocytes produce exosomes that carry several cargos, such as proteins and miRNAs (73). However, exosomes secreted from damaged hepatocytes are essential in inducing hepatocellular inflammation and fibrosis during liver damage. These effects are through intercellular communication between different cell types (74). The roles of exosomes and exosomal miRNAs in NAFLD pathogenesis and the interactions between different organs are illustrated in Figures 1, 2.

#### 4.1.2 Lipid accumulation

NAFLD is characterized by excessive hepatic lipid accumulation (75). The accumulation of lipids in the liver cells results in the release of stress signals, triggering the activation of inflammatory pathways that, when perpetuated, lead to chronic injury and fibrosis over time. Hepatocytes release EVs in response to lipotoxic fatty acids, which leads to HSCs fibrogenic activation and promotes macrophage chemotaxis (76, 77). Inflammation and fibrosis are essential for NAFLD progression (78).

Li et al. (79) demonstrated that the exosomal *miR-199a-5p* induces lipid build-up in the liver through a down-regulation of its target gene, hepatic Mammalian sterile 20-like kinase 1 (*MST1*). This leads to an alteration in Sterol regulatory element-binding protein 1 (SREBP1c), AMP-activated protein kinase (AMPK) signaling cascades and consequently suppresses Carnitine palmitoyltransferase I α (*CPT1*α) lipolysis gene and induces Fatty acid synthase (*FASN*) lipogenesis gene expression. In another study, Xu et al. (80) reported that hepatocyte exosomal *miR-34a* plays an essential role in the development of NAFLD by increasing lipid absorption and synthesis and reducing fatty acid oxidation. It also promotes NAFL's transition to NASH by regulating Kupffer cell activation, which promotes inflammatory responses, increases hepatic ROS levels, and induces hepatic apoptosis.

#### 4.1.3 Hepatocellular inflammation

Lipotoxic hepatocyte-derived EVs cause hepatocellular inflammation. NAFLD is closely related to chronic inflammation associated with macrophages and neutrophils (78). Additionally, innate immune activation is fundamental in triggering hepatic inflammation in NASH (81). Hirsova et al. (82) have demonstrated that incubation of primary hepatocytes with lysophosphatidylcholine (LPC) increased their EV secretion compared with control cells *in vitro*. EVs derived from hepatocytes, carrying TNF-related apoptosis-inducing ligand, activated *IL-1*β *and IL-6* messenger RNAs expression in macrophages derived from mice bone marrow, leading to inflammation and liver injury.

Regarding macrophage infiltration, Ibrahim et al. (83) have demonstrated that in hepatocyte lipotoxicity with LPC, Mixed lineage kinase 3 (MLK3) signaling induces the secretion of EVs containing elevated levels of CXCL10 from hepatocytes. These EVs induce bone marrow-derived macrophage chemotaxis. Moreover, Hepatocyte-derived ceramide-dependent EVs are enriched in several distinct damage-associated molecular patterns and cellular adhesion molecules, including ICAM1 and S100A11, that may influence immune cell responses, leading to hepatocellular inflammation (84). In addition, Kakazu et al. (77) have demonstrated that hepatocytes treated with palmitic acid (PA), a saturated fatty acid found in NAFLD hepatocytes, release significant amounts of C16:0 ceramide-enriched EVs in an inositol-requiring enzyme-1α (IRE1α)-dependent manner. IRE1A-stimulated hepatocyte-derived EVs, having ceramide-derived sphingosine 1-phosphate, enhance macrophage migration, which results in an inflammatory response in the liver (77, 85).

Moreover, in NAFLD, imbalanced macrophage polarization toward the pro-inflammatory M1 phenotype significantly contributes to disease progression (86). Conversely, M2 macrophages, particularly the M2a and M2c subtypes, exert protective effects in NAFLD (87). M1 macrophages are involved in the immune response and immune monitoring through antigen presentation and the release of pro-inflammatory cytokines like IL-1β and TNF-α (87). On the other hand, M2 macrophages have limited antigen presentation capabilities and contribute to immune regulation by dampening the immune response with the secretion of inhibitory cytokines such as IL-10, TGF-β, and Mrc (88). M2 macrophages can contribute to tissue healing and renewal. The decision of whether macrophages exhibit a pro-inflammatory reaction that causes injury or an anti-inflammatory response that offers protection depends on the balance between M1 and M2 activation tendencies (89).

Liu et al. (90) found that exosomal *miR-192-5p* and hepatocyte-derived exosome levels are considerably higher in NASH patients' serum, similar to high-fat, high-cholesterol diet (HFHCD)-fed rat NASH models. Progression of NAFLD in HFHCD-fed rats is directly associated with serum *miR-192-5p* and proinflammatory M1 macrophage amounts along with proinflammatory cytokines expression (90). Another study (91) showed that exosomes originating from lipotoxic hepatocytes and containing *miR-192-5p* significantly activate macrophages. They polarize macrophages to proinflammatory M1 phenotype by regulating rapamycin-insensitive companion of mammalian target of rapamycin (Rictor)/Akt/Forkhead Box Transcription Factor O1 (FoxO1) signaling pathway. In addition, saturated fatty acids and Cholesterol, via inducing lysosomal dysfunction, promote exosomal *miR-122-5p* secretion from hepatocytes. It results in pro-inflammatory M1 macrophage polarization and inflammatory activation (92).

The interaction between hepatic stellate cells (HSCs) and macrophages plays a crucial role in the pathogenesis of liver fibrosis (93, 94). Nevertheless, the specific molecular process that facilitates communication between hepatic stellate cells (HSCs) and liver macrophages, particularly the M2 subtype, remains incompletely understood. Exosomes are suggested to mediate the connection between mentioned cells, especially through the miRNAs (94). The polarization of M2 macrophages is strongly linked to the suppression of HSC activation and the reduction of liver fibrosis (95–97). Wan et al. (94) showed that HSC activity was significantly inhibited by exosomes derived from M2 macrophage. They found that exosomal *miRNA-411-5p* is reduced in the liver tissue and serum of HFHC diet-induced rat model of NASH. They also showed that exosomal *miR-411-5p* from M2 macrophages hinder the HSCs activity and lead to inactivation of stellate cells through downregulation of the expression of Calmodulin-Regulated Spectrin-Associated Protein 1 (*CAMSAP1*). The inhibition of HSCs occurred following the knockdown of *CAMSAP1* as a direct target of *miRNA-411-5p*. Furthermore, the amount of *miR-411-5p* in exosomes derived from M2 macrophages was higher than that in M1 macrophages. Recent literature shows that *MiR-411-5p* may play a role in the regulation of hepatocellular carcinoma progression (98).

Liu et al., (91) found that *arginase 1* (Arg1), a marker of M2 macrophages, was reduced after an 8-week high-fat high-cholesterol diet (HFHCD) in a rat model, but other M2 markers, *chitinase 3-like protein 3* (Ym1) and *found in inflammatory zone 1* (Fizz1), remained unchanged throughout the 16-week diet. Interestingly, they discovered that hepatocyte-derived exosomal miR-192-5p did not induce M2 macrophage polarization, and neither did exosomes from PA-treated HepG2 cells affect M2 macrophage activation. They also observed a decrease in Ym1 expression in THP-1 macrophages after exposure to serum exosomes from NASH patients, an effect that was reversed with a miR-192-5p inhibitor. In another study, Zhao et al. (92) investigated the potential role of cholesterol-loaded hepatocytes in inducing macrophage polarization through exosome-related cross-talk. They found that exosomes from Huh7 cells loaded with ox-LDL and MβCD-cholesterol did not affect the percentage of M2 macrophages. In conclusion, these studies suggest that while certain exosomes and exosomal miRNAs released by cholesterol-loaded hepatocytes can influence M2 macrophage polarization, their effects are complex and may depend on the specific conditions and markers examined. Further research is needed to fully understand these mechanisms and their implications in NAFLD. This could potentially open new avenues for therapeutic interventions targeting macrophage polarization. Changing the macrophage polarization in hepatic tissue toward M2 macrophages may exert protective effects against NAFLD progression and liver fibrosis. This should be further studied as a potential therapeutic pathway in NAFLD.

Besides, hypoxia can induce NAFLD in obstructive sleep apnea (OSA) syndrome by exosomes. Hypoxia triggers the production of certain exosomes (99). Yang et al. (100) mentioned that OSA-induced exosomes promote hepatocyte steatosis and activate macrophages in liver tissue. These exosomes were observed to enhance fat accumulation. They demonstrated that after the uptake of OSA-induced exosomes by macrophages, the polarization of macrophages toward the M1 type occurred. It led to the inhibition of sirtuin-3 (SIRT3)/AMP-activated protein kinase (AMPK) and autophagy. It also enhanced the activation of the nucleotide-binding domain, leucine-rich-containing family, and pyrin domain-containing-3 (NLRP3) inflammasomes. The use of 3-methyladenine (3-MA) to block autophagy prevented NLRP3 inflammasome activation and hindered M1 macrophage polarization. Moreover, they reported elevated levels of miR-421 in OSA-induced exosomes in OSA plus NAFLD mice and patients. In the liver tissues of OSA and OSA plus NAFLD mice, *miR-421* showed the same co-localization with macrophages. Hepatocytes exposed to intermittent hypoxia transferred *miR-421* to macrophages through exosomes to inhibit SIRT3 and contribute to macrophage M1 polarization. Accordingly, MiR-421 targeting reduced SIRT3 protein levels in macrophages. They also found that in *miR-421–/–* mice subjected to OSA and NAFLD modeling, liver steatosis and M1 polarization were notably reduced. Knockout of *miR-421* alleviated the inhibitory effects of OSA-induced exosomes on SIRT3 and autophagy, leading to reduced liver steatosis and macrophage M1 polarization. On the other hand, they haven't found any change in the transcription of M2 macrophage genes Arg1 and Fizz1 Following the uptake of OSA exosomes by macrophages.

Other than the above mechanisms, Shen et al. (101) showed that decreased hepatocyte autophagy, found in non-alcoholic steatohepatitis, develops IL-1β/TNF-induced hepatic injury and inflammation through the exosomal release of the damage-associated molecular pattern (DAMPs). In addition, liver inflammation could be caused by EVs secreted from other cells. For example, It has been reported that platelet-derived EVs may have a pro-inflammatory role in the liver (102). However, some studies have demonstrated the opposite (103).

#### 4.1.4 Fibrogenesis

Hepatic stellate cells (HSCs) promote fibrogenesis through EVs intracellular communication. Literature (104) has shown that hepatocytes stimulated by palmitate (PA) secret significantly more exosomes with distinctive miRNA expression patterns, amplifying fibrotic gene expression in HSCs. In this regard, Wei Wang et al. (105) illustrated that hepatocytes treated with PA release more *miR-107*-enriched exosomes, leading to HSC proliferation and activation by two distinct pathways. These hepatocyte-derived exosomes transfer *miR-107* to HSCs, where *miR-107*, by directly inhibiting Dickkopf-1 (*DKK1*) expression, activates Wnt signaling. Additionally, these exosomes deliver *miR-107* to CD4^+^ T cells, where *miR-107* activates the Raf/MEK/ERK signaling pathway by upregulating *IL-9* expression via Forkhead box protein P1 (*Foxp1*) inhibition. These pathways lead to HSC activation, an essential component of NAFLD pathogenesis.

On the other hand, exosomes may exert protective effects on NAFLD. Studying on a diet-induced rat model of NASH, Qi et al. (106) proved that M2 macrophage-derived exosomes enriched with *miR-411-5p* directly reduce Calmodulin-Regulated Spectrin-Associated Protein 1 (*CAMSAP1*) expression. This consequently inhibits hepatic stellate cell activation and fibrosis. In addition, Hou et al. (107) found that *miR-223*-enriched exosomes are released from myeloid cells in response to IL-6 signaling. They offer protective effects on NASH progression by inhibiting the expression of profibrotic genes that *miR-223* targets, including C-X-C motif chemokine (*Cxcl10*), NOD-, LRR—and pyrin domain-containing protein 3 (Nlrp3), transcriptional activator with PDZ-binding (Taz), and insulin-like Growth Factor 1 Receptor (*Igf1r*) in the liver.

#### 4.1.5 Adipocyte-hepatocyte interaction

Adipocyte-hepatocyte interaction through EVs plays an essential role in NAFLD progression. Afrisham et al. (108) demonstrated that plasma exosomes isolated from obese women may play a role in NAFLD development by inducing insulin resistance, increasing hepatocellular TG levels, and decreasing hepatocellular FGF21 secretion. These exosomes promote a significant increase in the expression of *integrin* α*νβ-5* and tissue inhibitor of matrix metalloproteinase-1 (*TIMP-1*) along with a concomitant decrease in plasminogen activator inhibitor-1 (*PAI-1*) and matrix metalloproteinase-7 (*MMP-7*) expression in HepG2 cells. In hepatic stellate cells, these exosomes induce increased *integrin* α*νβ-5 and 8, Smad-3, TIMP-1* and *4*, and matrix metalloproteinase-9 (*MMP-9*) expression. Consequently, these processes dysregulate the tumor growth factor- β (TGF-β) signaling pathway, producing a profibrotic state in liver cells (109).

According to the tissue-cooperative homeostatic model of NAFLD, during the early phases of NAFLD progression, the falling levels of the hepatocellular production of miR-122 are compensated by an increase in the adipose miRNA-containing exosome secretion (110). These molecules are eventually taken up by the hepatocytes and augment these cells' endogenous production of miRNA. Thus, metabolic damage to adipose tissue (due to liver disease progression) eventually decreases the external supply of liver-supporting miRNAs, leading to hepatic fibrosis and carcinogenesis.

Ogur et al. (111) determined that exosomes derived from Endoplasmic Reticulum (ER) stress-induced adipocytes transfer exosomal Aldo-keto-reductase 1b7 (Akr1b7) to hepatocytes. This elevates glycerol levels in liver cells, which gives rise to hepatic steatosis, inflammation, and eventually fibrosis. Additionally, Yan et al. (112) showed that PA-induced AMPKα1 inhibition in adipocytes promotes CD36-containing exosome secretion. These exosomes contribute to HepG2 cell damage and mediate the development of High-fat diet (HFD)-induced NAFL. Furthermore, exosomal *let-7b-5p* originated from hepatocytes is essential in the interaction between hepatocytes and adipocytes by TGF-β signaling. It impairs the energy balance of adipocytes by regulating *Adrb3* gene expression, resulting in hepatic steatosis and obesity (113). Besides, Fuchs et al. (114) found that subcutaneous abdominal adipose tissue-derived exosomes and raised levels of PAI-1 take part in the pathophysiology of insulin resistance. Insulin resistance is a critical factor in the NAFLD pathobiology (115).

Exosomes of adipose tissue may also have beneficial effects on NAFLD. Zhao et al. (116) demonstrated that exosomes from adipose-derived stem cells (ADSCs) promote arginase-1 expression in macrophages by carrying active STAT3 and inducing M2 phenotype macrophage polarization. This results in the inhibition of macrophage inflammatory responses. It suggests the potential efficacy of exosome and stem cell therapy in preventing NAFLD progression.

#### 4.1.6 Other pathological pathways

EVs also contribute to the development of NAFLD through other pathophysiological mechanisms. In a study on *Asc* ^−/−^ mice on a high-fat diet (HFD), Chen et al. (117) revealed a gut-liver axis mechanism in which exosomes play a significant role. In dysbiosis, exosomes act as the transporter of high mobility group box 1 (HMGB1) protein from the intestine to the liver, triggering hepatic steatosis. Besides, a preliminary study (118) on TSH-induced lipotoxicity in NAFLD revealed that TSH stimulation of HepG2 cells significantly increases their exosomal production and alters their exosomal proteomic profile. It leads to upregulation of proteins involved in different biological processes such as metabolism, inflammation, and apoptosis. Hence, it may be involved in NAFLD pathogenesis.

#### 4.1.7 Exosomal micrornas

miRNAs are the most abundant cargo molecules transferred by exosomes (119). Specific miRNAs have been associated with NAFLD and may be effectual in its pathogenesis. They may also be used as non-invasive options in NAFLD diagnosis. Zhang and Pan (120) monitored serum exosomal microRNAs in children with NAFLD and identified 2,588 miRNAs. They revealed that in children with NAFLD, the expression of 80 miRNAs, importantly *miR-122-5p, miR-335-5p*, and *miR-27a*, differs from that of the control group. In another study, Zhou et al. (121) suggested that exosomal miRNAs might take part in the pathophysiology of NAFLD and revealed an upregulation of *miR-146b-3p, miR-155-5p, miR-122-5p*, and *miR-34a-5p* in NAFLD patients. *MiR-122*, a highly liver-specific miRNA, takes part in lipid metabolism and is detected in the form of exosomes in the serum of NAFLD patients (122, 123). *MiR-21* is another miRNA that is increased in the liver of NAFLD patients, as well as animal models of the disease. This miRNA regulates hepatocellular glucose and lipid metabolism. It works through a complex transcription network. At different stages, *miR-21* may be involved in NAFLD progression, including early steps of the initiation of hepatocellular steatosis and later steps of inflammation and fibrosis (124). A list of exosomal miRNAs that may be beneficial for NAFLD diagnosis is presented in Table 1.

**Table 1:** Potential exosomal microRNAs in NAFLD diagnosis.

**microRNA**	**Associated tissue**	**Associated condition**	**Alteration of microRNA levels**	**Target gene/protein**	**Pathogenesis**	**References**
*miR-21*	Serum	NAFLD	Increased	*PPARα*	Dysregulating lipid metabolism in the liver	(125)
				*FABP7*	Dysregulating the transporting and metabolizing fatty acid	(126)
				*HMGCR*	Producing cholesterol and isoprenoids	(127)
				*HBP1*	Dysregulating the cell cycle, transcriptional repressor	(128)
		NASH	Increased	*SMAD7*	Suppressing the Inhibition of the TGF-β signaling	(129)
		Liver fibrosis	Increased	*TIMP-3*	Inhibiting the matrix metalloproteinases	(130)
*miR-122*	Serum	NAFLD	Increased	*SIRT1*	Suppressing the regulation of hepatic lipid metabolism Promoting hepatic oxidative stress Promoting hepatic inflammation	(131, 132)
*miR-27a*	Liver	NAFLD	Increased	*FASN* and *Scd1*	Attenuating hepatic *de novo* lipogenesis Alleviated obesity-initiated NAFLD	(133)
		MAFLD		*PINK1*	Inhibiting mitophagy Promoting MAFLD-related liver fibrosis	(134)
*miR-155-5p*	Liver	NAFLD	Increased	*STC1*	Inducing hepatic mitochondrial dysfunction, vascular insulin resistance Promoting endothelial cell activation and atherosclerosis	(135–137)
*miR-34a*	Serum	NASH NAFLD	Increased	*PPARα* and *SIRT1*	Hepatic lipid accumulation	(138)
*miR-192*	Serum	NAFLD	Decreased	*SREBF1*	Dysregulating lipid homeostasis in hepatocytes	(139)
*miR-181a*	Serum	NAFLD	Increased	*SIRT1*	Reducing insulin sensitivity Increasing gluconeogenesis and lipid synthesis	(140)
*miR-29a*	Serum	NAFLD	Decreased	*GSK3β*	Promoting of mitochondrial proteostatic stress	(141)
*miR-199a-5p*	Adipose tissue	NAFLD	Increased	*MST1*	Aggravating lipid accumulation in hepatocytes	(79)

PPARα, Peroxisome proliferator-activated receptor alpha; FABP7, Fatty Acid Binding Protein 7; HMGCR, 3-Hydroxy-3-Methylglutaryl-CoA Reductase; HBP1, HMG-Box Transcription Factor 1; SMAD7, SMAD Family Member 7; TIMP-3, Tissue inhibitors of metalloproteinases 3; SIRT1, Sirtuin 1; FASN, Fatty acid synthase; SCD1, Stearoyl-CoA Desaturase-1; PINK1, PTEN-induced kinase 1; STC1, Stanniocalcin 1; SREBF1, Sterol regulatory element-binding protein-1; GSK3β, Glycogen synthase kinase-3 beta; MST1, Macrophage Stimulating 1; MAFLD: Metabolic dysfunction-associated fatty liver disease.

### 4.2 Exosomes in NAFLD diagnosis

Studies have shown that exosomes, due to their contents, including microRNAs, may serve as potential diagnostic biomarkers for disease progression and severity in NAFLD (142). The content of exosomes can change in various diseases, and this is not only limited to exosomal miRNAs. As mentioned earlier, several exosomal miRNAs are considerably altered in NAFLD, and their potential role in NAFLD pathophysiology remains an important topic for a better understanding of NAFLD development and the mechanisms that help us ameliorate the condition. Meanwhile, the modification of the level of these miRNAs may provide a fruitful non-invasive diagnostic approach for NAFLD, considering the challenges in diagnosing NAFLD. A list of exosomal miRNAs potential for NAFLD diagnosis is presented in Table 1. Moreover, various studies have shown that changes in the level of exosome contents other than miRNAs, including protein FZD-7, can also act as diagnostic and prognostic biomarkers for NAFLD (143, 144). In addition to the content of exosomes, the level of certain EVs in mouse models and human subjects of NASH has been reported to be higher than that of normal individuals, suggesting specific exosomes' levels as potential factors for differentiating the stages of NAFLD (145). However, although exosomes offer favorable non-invasive methods for the diagnosis and prognosis evaluation of NAFLD, there is a lack of comprehensive information regarding the sensitivity and specificity of exosome-based diagnostic methods. There is a need for further investigation to understand better the diagnostic capabilities of exosomes and their practical application in clinical settings for NAFLD assessment.

### 4.3 Exosome-related strategies for NAFLD treatment

**Figure 3:**
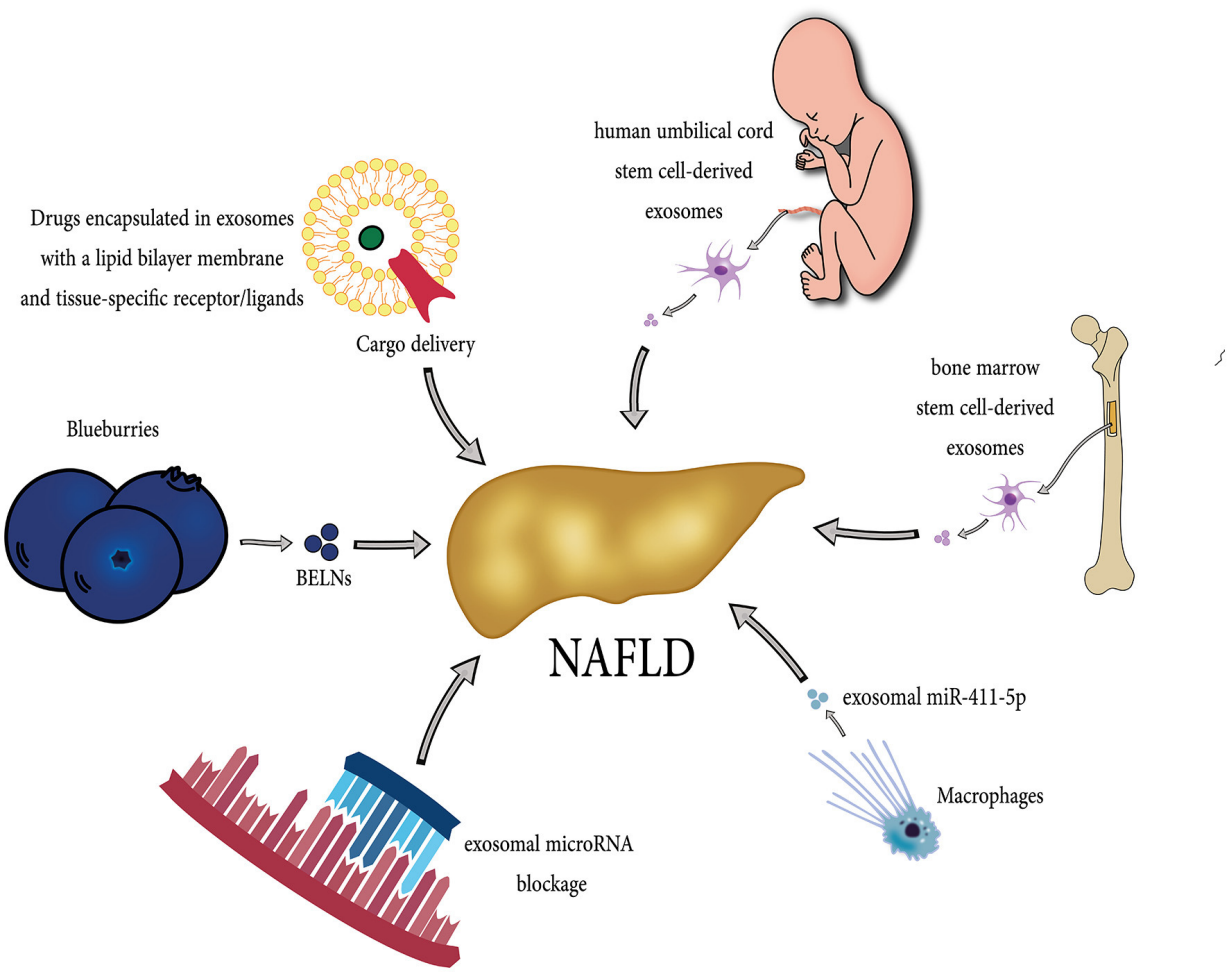
Diverse potential exosome-based therapeutic strategies in NAFLD. Exosomes originating from different sources, including stem cells, bone marrow, macrophages, and Blueberries, can exert beneficial effects in the treatment of NAFLD. Each source contributes unique therapeutic properties, such as anti-inflammatory and regenerative capabilities, immune modulation, and fibrosis inhibition, making them a promising tool in NAFLD management. They can be administered safely in high doses due to their cell-free nature, exhibit low toxicity, remain stable during circulation, and have minimal immunogenicity. Moreover, researchers are actively exploring exosome-based therapies for NAFLD, including drug delivery (serving exosomes as drug carriers, delivering therapeutic cargo directly to liver cells), targeted approaches (blocking exosomal contents such as microRNAs), and modulation of inflammatory processes.

Resmetirom has been recently approved by the Food and Drug Administration (FDA) and remains the only approved option for NAFLD treatment along with lifestyle modifications, such as diet control and exercise. However, several drugs have shown potential therapeutic effects on NAFLD, such as elafibranor (by inhibiting lipid deposition), emricasan (via lowering cell death), IMMe124 (through regulating intestinal microenvironment and metabolism) (146). Moreover, Probiotics and prebiotics, symbiotic, fecal microbiome transplantation, and fasting-mimicking diet could also be beneficial in patients with NAFLD (147). Due to their unique characteristics, exosomes have gained attention as a new treatment option in different diseases. Exosomes have been studied for various clinical applications, including as a diagnostic/prognostic biomarker, cell-free therapeutic agent, drug delivery carrier, and cancer vaccines (148). Several exosome-based treatment approaches have shown efficacy in liver diseases, including NAFLD, which is described below. Figure 3 demonstrates potential exosome-based treatment approaches in NAFLD.

#### 4.3.1 Cell-derived exosomes

In recent years, interest in therapeutic usage of cell derivatives such as exosomes over cell therapies has been increasing (149, 150). Exosomes are released by the majority of body cells and have been detected in nearly all fluids of the body, such as blood, cerebrospinal fluid, urine, amniotic fluid, breast milk, saliva, bile, malignant ascites, and lymph (151–158). Among all cell types, Mesenchymal stem cells (MSCs) are the most prolific producer of exosomes (159). MSCs can be derived from different body tissues, including peripheral blood, adipose tissue, bone marrow, umbilical cord, spleen, liver, pancreas, kidney, lung, thymus, and brain (160, 161). They are known as a subgroup of stromal stem cells. MSCs are adults' most researched type of stem cells in regenerative medicine owing to their extensive presence in body tissues and easy expansion procedure *in vitro*. They have shown the potential to prolong and save lives (162). Mesenchymal stem cell therapy has several disadvantages that must be considered, including the possibility of tracking in pulmonary capillaries, leading to pulmonary embolism, reduction in cell viability of MSCs in cryogenic storage during transport, and the risk of tumorigenesis after administration (163–166).

Exosomes mediate intracellular communication by transferring a variety of biomolecules like microRNAs (miRNAs), messenger RNAs (mRNAs), other non-coding RNAs, and lipids (167). Exosomes derived from stem cells have demonstrated the same therapeutic potential as their parental cells (162). It has been indicated that mesenchymal stem cell-derived exosome can alleviate liver fibrosis, decrease Alanine aminotransferase (ALT) and Aspartate transaminase (AST) levels, and mitigate liver inflammation (168). Thus, these exosomes, such as HUC-MSCs-derived exosomes, can be a promising therapeutic for NAFLD.

##### 4.3.1.1 HUC-MSC exosomes

Many reports have illustrated that human umbilical cord mesenchymal stem cells (HUC-MSCs) exert therapeutic effects on liver diseases, including chemical-induced liver injury, decompensated liver cirrhosis, liver failure, and autoimmune liver diseases, including primary biliary cholangitis (169–174).

###### 4.3.1.1.1 Lipid and glucose metabolism

HUC-MSCs improve NAFLD and metabolic syndrome by means of regulating lipid metabolism by promoting the expression of genes that are related to fatty acid oxidation and suppressing adipogenesis-related genes' expression in db/db mice (175). MSCs exert their therapeutic effects through secretory factors, including exosomes. Cheng et al. (176) showed that the treatment of palmitic acid (PA)-treated human normal liver cell line (L-O2 cells), human fetal hepatocyte line, with HUC-MSCs-exosomes improves cell viability and inhibits apoptosis. They showed that the level of *miR-627-5p* was higher in HUC-MSCs-exosomes compared with HUC-MSCs. Moreover, they found that the expression of G6Pc and PEPCK, gluconeogenesis-related proteins, along with FAS and SREBP-1c, lipid metabolism-related proteins, were repressed in these cells, while the expression of PPARα, another lipid metabolism-related protein, was notably downregulated. Additionally, the study exhibited that the plasma levels of ALT, AST, total cholesterol (TC), triglyceride (TG), and blood glucose in the NAFLD rat model were repressed by exosome treatment. They also reported that *MiR-627-5p* improves lipid and glucose metabolism, key pathological components of NAFLD, in L-O2 cells by targeting fat mass and obesity-related gene (*FTO*). The *FTO* gene facilitates NAFLD development via increasing insulin resistance, oxidative stress, and lipid accumulation in liver cells (177, 178). Furthermore, exosome treatment has also been found to alleviate insulin resistance and liver damage and reduce fat accumulation in NAFLD rat models (176). Another study (179) also confirmed that HUC-MSC-exosome therapy can attenuate liver steatosis and regulate abnormal expression of *Fabp5, ACOX, PPAR-*α, *FAS, SREBP-1c*, and *CPT1*α as lipid metabolism-related genes.

###### 4.3.1.1.2 Oxidative stress

HUC-MSC-Exosomes have also been shown to have beneficial effects against oxidative stress and inflammation. Kang et al. (179) demonstrated that in rat models of NASH, HUC-MSCs exosomes enhanced the Nrf2 (Nuclear factor erythroid 2-related factor 2), a protective factor against oxidative stress and 1 [NAD (P) H quinone dehydrogenase 1], a part of cellular adaptive response to stress, which seems to play a significant part in treating NASH (180). Besides, HUC-MSC-derived exosomes reduce oxidative stress through lowering Malondialdehyde (MDA), CYP2E1, and reactive oxygen species (ROS) levels and elevating Superoxide dismutase (SOD) and GSH function as well (179). Furthermore, HUC-MSC-exosome therapy reduces inflammatory response through decreasing F4/80^+^ and CD11c^+^ macrophages and the levels of tumor necrosis factor-alpha (TNF-α) and Interleukins-6 (IL-6) (179).

##### 4.3.1.2 BM-MSC exosomes

MSCs are classically separated from bone marrow (181). Bone marrow mesenchymal stem cells (BM-MSCs), also known as BM mesenchymal stromal cells, are multipotent mesenchymal precursor cells that have many favorable characteristics for regenerative therapy, including anti-inflammatory and immune-modulatory properties (182, 183). Studies have revealed that BM-MSCs have therapeutic potential in different diseases, including cardiovascular, lung, neural, hematopoietic, and liver diseases, along with graft-vs.-host disease and cutaneous, tendon ligament, and musculoskeletal tissue repairing (184–186). BM-MSCs release paracrine factors which influence the surrounding microenvironment (187). In contrast with their favorable therapeutic characteristics, a few downsides can restrain their clinical utilization, primarily due to their potential for tumorigenicity and immunogenicity (188). Thus, using BM-MSCs paracrine factors maintains BM-MSCs properties without most disadvantages. Therefore, exosomes can potentially replace BM-MSCs as a safer therapeutic approach for tissue repair (162, 189).

BM-MSCs and BM-MSC-derived exosomes both can provide anti-steatotic effects through downregulating sterol regulatory element binding protein 1 (*SREB-1*), sterol regulatory element binding protein 2 (*SREB-2*) and cetyl coenzyme A carboxylase (*ACC*), suppressing lipid uptake and upregulating peroxisome proliferator-activated receptor alpha (*PPAR-*α) and carnitine palmitoyltransferase 1 (*CPT1*) fatty acid oxidation genes. BM-MSC-derived exosomes at 15 μg/kg, 30 μg/kg, and 120 μg/kg have anti-steatotic effects in the HFD-induced NASH model. BM-MSCs or BM-MSCs-exosome co-treatment caused anti-apoptotic impacts via a meaningful reduction in *Bax/Bcl2* ratio and a rise in the expression of mitochondrial mitophagy genes, including Parkin RBR E3 Ubiquitin Protein Ligase (*Parkin*), phosphatase and tensin homolog (PTEN)-induced putative kinase 1 (*PINK1*), unc-51 like autophagy activating kinase 1 (*ULK1*), B-cell lymphoma 2/adenovirus E1B 19 kDa protein-interacting protein 3 (*BNIP3L*), autophagy related gene (*ATG5*), *ATG7* and *ATG12*. Furthermore, a notable depletion in the levels of AST and ALT has been observed in BM-MSCs or BM-MSCs-exosome co-treatment (190).

El-Derany et al. (190) found that BM-MSC-derived exosome can be an ideal treatment option for NAFLD through a mechanism in which the upregulation of *miRNA-96-5p* leads to the inhibition of *caspase-2*. *MiRNA-96-5p* is found both in peripheral blood and bone marrow (191). Studies have demonstrated that HFD is associated with a downregulation of *miRNA-96-5p* in NAFLD models (190, 192, 193). Moreover, it has been proven that the suppression of *caspase-2* lowers lipo-apoptosis and inhibits fibrogenic Hedgehog ligands production, leading to the transition of NAFL to Nash (190, 194, 195). Furthermore, evidence has revealed that the inhibition of *caspase-2* prevents hyperlipidemia and reduces hepatic steatosis, liver apoptosis, and mitochondrial mitophagy (190, 194).

##### 4.3.1.3 Macrophage-derived exosomes

Severe stages of NAFLD are related to hepatic stellate cell (HCS) activation and their interactions with macrophages. Hepatic macrophages have critical roles in the initiation and progression of fibrosis (93, 196, 197). It has been found that the level of *miRNA-411-5p* is lower in NASH patients' plasma exosomes and liver samples of NAFLD patients (94). Moreover, M2 macrophage-derived exosomes contain a higher amount of miRNA-411-5p than M1 macrophages. Wan et al. (94) revealed that M2-derived exosomes suppress HCS activation by inhibiting the direct target of *miRNA-411-5p*, Calmodulin-Regulated Spectrin-Associated Protein 1 (*CAMSAP1*), during the transition of NASH to NAFL.

In macrophages and neutrophils, *miR-223* is the amplest miRNA that is functionally active and can be transferred from myeloid cells to other cells by exosomes (198–200). The serum level of *miR-223* was reported to be remarkably higher in NAFLD. Moreover, a positive correlation has been seen between the levels of serum *miR-223* and IL-6 (107). IL-6 signaling exerts anti-fibrotic effects in NAFLD-associated liver fibrosis by inducing myeloid cells to enhance *miR-223*-enriched exosome release. IL-6 increases the biogenesis of exosomes by upregulating gene expression without changing the *pre-miRNA-223* expression (107). Myeloid cell-derived *miRNA-223* enriched exosomes are delivered to the liver and inhibit the expression of *miR-223* target pro-fibrotic genes like *Igf1r, Cxcl10, Taz*, and *Nlrp3*. Subsequently, they help control Nash's progression. Therefore, macrophage-derived exosomes could be used as a therapeutic option that alters the progression of NASH to NAFL (107).

#### 4.3.2 Exosome blocking

Exosomes are the communication bridge between cells, thus playing critical roles in the pathological development and progression of numerous diseases (201). Accordingly, changing the level of exosome secretion, inhibiting their activity, or changing their contents could be a potential approach for treating diseases.

##### 4.3.2.1 Exosomal mirna blocking

Targeting exosomal miRNAs may also be an effective treatment approach in NAFLD. miRNAs are reported to have therapeutic roles in NAFLD by regulating lipid metabolism, inflammation, and fibrosis (90). As mentioned in the pathophysiology part, exosomal miR-192-5p is higher in exosomes derived from NASH hepatocytes and plays a role in NAFLD progression by triggering M1 macrophage polarization (90). It has been found that this mechanism can be reversed using a *miR-192-5p* inhibitor (91). Hence, targeting serum exosomal *miR-192-5p* can be a beneficial option for inhibiting the progression of NAFLD (91).

Moreover, *MiR-122-5p* is another prospective option in exosome blocking. Zhao et al. (92) demonstrated that *miR-122-5p* level was higher in exosomes originating from Huh7 cells, a hepatic cell line loaded with cholesterol (including ox-LDL and MβCD-cholesterol). They used *anti-miR-122-5p* to block this exosomal miRNA. *Anti-miR-122-5p*-treated Huh7 cells-derived exosomes exerted lower effects in inducing TNF-α, IL-6, and IL-1β expression. Additionally, exosome-mediated inflammation of macrophages was blocked.

##### 4.3.2.2 Blocking liver-adipose tissue crosstalk

Adipose tissue exosomes may also be favorable therapeutic targets for NASH. Several studies have supported the role of pro-inflammatory mediators released from visceral adipose tissue (VAT) in the progression of NAFLD (202, 203). A cross-talk between adipose tissue and the liver is significant in NAFLD development and progression. This connection seems to be facilitated through exosomes (204). Stressed AT induces abnormal lipid accumulation in the liver, which may be the major contributor to NASH initiation and progression.

Studies have indicated that NASH development can be a consequence of Endoplasmic Reticulum (ER)-stress, which possibly changes miRNAs and metabolites of secretory exosomes (77, 205). Exosomes derived from ER-stress induced-AT can induce and aggravate NASH by delivering exosomal Aldo-keto reductase family 1 B7 (AKr1b7) (111). They can be taken up by hepatocytes and cause hepatic steatosis, inflammation, and fibrosis. Akr1b7 deficiency protects murine liver from NASH in HFD and methionine-choline-deficient diet (MCD)-fed mice. In addition, suppressing hepatic AKr1b7 reduces TG level and glycerol concentration and alleviates hepatic inflammation, ER stress, and lipid synthesis. Accordingly, Epalrestat, an Akr1b7 inhibitor, can suppress the effects of AT exosomes on ER stress and hepatic inflammation (68). Moreover, Treatment with GW4869, an exosome production inhibitor (by inhibiting sphingomyelinase), has been shown to alleviate ER stress and reduce lipid synthesis and hepatic inflammation (111).

##### 4.3.2.3 TGF-B signaling blockage

TGF-B signaling plays a major part in various biological processes, such as cellular proliferation, differentiation, migration, and cell death. It is also an essential pathway in the regulation of liver homeostasis. TGF-B signaling helps NAFLD progression from initial lipid accumulation to fibrosis (206). Steatosis, weight gain, and impaired insulin sensitivity have been found to be associated with TGF-B signaling in a mouse model of NASH (207). Moreover, TGF-B signaling can modulate microRNA biogenesis at transcriptional and post-transcriptional levels (208). TGF-B induces hepatic secretion of exosomes carrying *let-7b-5p*, a miRNA. TGF-β-let-7b-5p pathway contributes to HFD-induced steatosis and obesity through lowering oxidative phosphorylation in mitochondria and inhibiting white adipose tissue (WAT) to brown fat conversion (113). Accordingly, *Tgfbr2* loss in hepatocytes ameliorates HFD-induced NAFLD by improving mitochondrial biogenesis. It alleviates lipid accumulation and induces “browning” of WAT via inhibiting miRNA *let-7b-5p*-containing exosomes release from hepatocytes. In addition, *let-7b-5p* inhibitor can downregulate the expression of *Cd36, Fatp1*, and *Fabp1* (which are fatty acid transporter genes), upregulate mitochondrial genes' expression, like *Cox5b* and *Atp5a*, and reduce lipid accumulation in CL-316,243 (CL)/cold-exposed mice and HFD-induced obese mice (113).

##### 4.3.2.4 Drugs blocking exosomes

Some drugs are also effective in exosome blockage and provide benefits through exosome modulation. GW4869, a non-competitive neutral sphingomyelinase inhibitor, suppresses exosome secretion. It has been shown to mitigate HFD-induced NAFLD in mice models (112). Moreover, McCommis et al. (209) evaluated the efficacy of next-generation thiazolidinediones (MSDC-0602) in a mouse model of NASH. They have weak binding to peroxisome proliferator-activated receptor γ (PPARγ) and still directly suppress and interact with mitochondrial pyruvate carrier (MPC). The effect of MSDC-0602 was through MPC2. They revealed that MSDC-0602 treatment, directly and indirectly, prevents and reverses stellate cell activation, liver fibrosis, and lipid accumulation in HTF-C mice. It is through the indirect modulation of exosomes derived from hepatocytes in an MPC-dependent manner. Furthermore, Yan et al. (112) found that metformin treatment inhibits HFD-induced NAFLD in mice, thorough activating AMP-activated protein kinase (AMPKα1) in WAT, which leads to the inhibition of exosomes shedding into serum and WAT.

Ezetimibe is a lipid-lowering drug that selectively inhibits the absorption of cholesterol through the blockage of NBC1LI-dependent cholesterol transport in the small intestine. NBC1LI is also present in the liver (210–212). Many studies have demonstrated that ezetimibe treatment can significantly improve hepatic steatosis, ballooning score, and NAFLD histological features, even after its fibrotic changes (213, 214). It has been reported that ezetimibe treatment activates autophagy in human hepatocyte cells (215). In addition, Kim et al. (216) observed other beneficial effects of ezetimibe in NAFLD. They reported changes in Hepatocyte-macrophage interaction following ezetimibe treatment. They observed decreased Interleukin 1 Beta (IL-1B) mRNA and protein levels in macrophages cultured with EVs released from ezetimibe/PA-cotreated hepatocytes.

#### 4.3.3 Cargo delivery

Recently, a variety of different nano-based drug carriers have been used in order to improve the therapeutic efficacy of chemical and biomolecular drugs and ingredients (217). Exosomes have favorable characteristics for being used as a drug delivery agent, including small size, which facilitates its penetration into deep tissues (218), having a slightly negative zeta potential (219), biocompatibility, low immunogenicity, being able to cross the biological barriers such as the BBB (220) and escape immune clearance (221), low systemic toxicity, and long-term existence in the target tissue (221, 222). Moreover, compared to many synthetic drug delivery agents, using exosomes facilitates cellular uptake due to their specific surface proteins (218, 223).

The exosomal-based delivery system has been loaded with a variety of biomolecules for drug delivery like paclitaxel, doxorubicin, curcumin as a peptide or protein-based therapeutics containing STAT3 inhibitors18, catalase, and also genetic material including siRNA (222), an effective carrier of miRNA that also stabilizes the miRNA is exosome (224, 225).

#### 4.3.4 miRNA-based therapeutics

MiRNAs are non-coding RNAs that take part in the regulation of gene expression. Recently, some studies have revealed that miRNA-based therapies are feasible and effective in various diseases, especially cancers (226–228). For instance, *miR-16*-loaded minicell is in clinical investigation for the treatment of malignant pleural mesothelioma (229). MiRNAs may be potential treatment options for NAFLD as well. In the early stages of NAFLD, decreased intrahepatic miRNA levels are compensated by the elevated production of miRNA in adipose tissue. As NAFLD progresses, the external supply of liver-supporting miRNA falls gradually, which leads to the deterioration of liver function and an increase in hepatic carcinogenesis (110). Therefore, supplying damaged liver with external miRNA may be efficient in hindering the progression of NAFLD. Moreover, Baranova et al. (110) revealed that purified *miR-122* exosomes may be potential cell-free therapeutics for preventing HCC in NAFLD. Further research on the efficiency of miRNA therapy in NAFLD is needed.

Blocking pathogenic exosomal miRNAs is also a potential treatment approach for NAFLD. The level of *miR-199a-5P* is found to be considerably higher in NAFLD. Exosomal *miR-199a-5P* (Exo-miR-199a-5P) interferes with the metabolism of lipids in the liver by suppressing the expression of hepatic MST1 (79). Additionally, administration of Exo-miR-199a-5P induces hepatic lipid deposition in normo-caloric diet (NCD) and HFD mice (which is possibly via regulating AMPK and SREBP-1c signaling pathways) (79). It also elevates liver weights and plasma TG levels (79). Expectedly, exosomal *anti-miR-199* can significantly reduce lipid accumulation *in vivo* and *in vitro*. It also downregulates lipogenic genes and lowers serum and hepatic TG and cholesterol in HFD mice (79). Thus, treatment with exosomal *anti-miR-199a* can offer fruitful effects in the prevention and treatment of liver steatosis and dyslipidemia.

#### 4.3.5 Exosomes-like nanoparticles

Plant-derived exosome-like nanoparticles (PDNPs) have shown potential in drug delivery and as therapeutic applications in different disease (230, 231). PDNPs are nano-sized vesicles released from edible plants, including lemon, apple, carrot, grapefruit, coconut, broccoli, grape, and ginger (232–241). Increasing evidence indicates that similar to mammalian cell-secreted exosomes, PDNPs have an important in intercellular communication (242, 243). In addition, they have certain superiorities compared to mammalian cell-secreted exosomes or artificial nanoparticles; they do not cause any detectable Immunogenicity or toxicity, they have high potential in delivering biomolecules, and using them is highly economical (95, 107, 110). They have been used in treating alcoholic fatty liver as well as IBD and colon cancer (234, 237, 244–246).

Blueberry nanoparticles have shown promise in the treatment of NAFLD. Blueberry is one of the most consumed berries that contains a wide diversity of bioactive compounds, such as flavonoids, anthocyanins, and polyphenols. Blueberry has antioxidative, anti-inflammatory, and anti-tumor activity and can have protective effects against cancers as well as Alzheimer's cardiovascular diseases, and depression (247–251). Zhao et al. (252) investigated the anti-oxidative impacts of Blueberry-derived exosomes-like nanoparticles (BELNs) in hepatocytes. They demonstrated that BELNs could attenuate oxidative stress by modulating nuclear factor erythroid 2–related factor 2 (Nrf2) distribution, which results in the upregulation of the antioxidative proteins and enzymes expression such as SOD, NADPH/quinone oxidoreductase I (NQ01), Heme Oxygenase-1 (HO-1), glutathione peroxidase (GPx), and catalase (CAT). It also regulates Bcl-2-associated X protein (BAX), Bcl-2, and HO-1 apoptosis-related proteins' expression in the liver of HFD-fed mice and rotenone-induced HepG2 cells. BELN supplementation also improves liver dysfunction and insulin resistance in NAFLD, which is accompanied by a reduction in AST and ALT levels and fat deposition as well as a downregulation in acetyl-CoA carboxylase 1 (ACC1) and fatty acid synthase (FAS) expression (252).

#### 4.3.6 Exosome-melatonin cotreatment

Melatonin can potentiate the therapeutic effects of exosomes; thus, it may be a favorable agent to be administered alongside exosomes. Melatonin is a methoxyindole that is secreted by the pineal gland and gastrointestinal tract during night and daytime, respectively (253). Melatonin has a critical role in the amelioration of different metabolic disorders in various tissues, including adipose and hepatic tissues (254–256). Melatonin exerts its beneficial effects in NAFLD exosomal therapy by activating Bmal1 expression in adipocytes (257). Eventually, Exosome-Melatonin co-treatment alleviates ER-stress-induced hepatic steatosis through a significant downregulation in the expression of adipocyte-derived exosomal resistin (257). Resistin is an adipocytokine that may aggravate hepatic steatosis via stimulating hepatic ER stress. Furthermore, exosome-melatonin supplementation can lower hepatic inflammation, fibrosis, and cell apoptosis (257).

### 4.4 Challenges and opportunities

The growing prevalence of NAFLD and its global health burden have provoked considerable research on possible diagnostic and therapeutic options for NAFLD. NAFLD is a multifactorial disease which includes a broad spectrum of liver damage. Although various pathophysiological mechanisms and genetic factors have been proven to be related to NAFLD, its treatment remains challenging. In recent years, exosomes have attracted widespread attention for their role in metabolic dysfunctions and their efficacy as pathological biomarkers. Exosomes have also shown tremendous potential in treating various disorders, such as wound healing and neurological and cardiovascular dysfunctions. With increasing evidence supporting the significant role of exosomes in NAFLD pathogenesis, their theragnostic potential has become a point of interest in NAFLD. Considering the low cost and high feasibility of EV detection and its non-invasiveness, using exosomes as a diagnostic biomarker can be a revolution in NAFLD diagnosis. Exosome-based therapies can also bring favorable results in preventing and treating NAFLD. However, there are still certain limitations that need to be overcome, and many questions need to be answered before exosomes can be utilized as a routine treatment for liver diseases.

Contrary to the satisfactory results of experimental exosome therapy studies, exosomal therapy methods and safety are not entirely clarified (258). Exosome-based therapy methods face both pharmaceutical (production, isolation, and drug loading) and pharmacokinetic (biodistribution and cellular uptake) challenges in becoming clinical. One of the main limitations of exosome therapy is the preparation and purification of a large volume of desired exosomes. Moreover, designing standard methods to isolate and identify the types and sources of exosomes also needs to be investigated more since the purity and physicochemical properties of exosomes are strongly affected by their isolation method, and no globally accepted standard is currently available in this era. Besides, considering exosomes' degradation during isolation and freeze-thaw processes, standardization of isolation and storage conditions is also crucial (148, 259–261). Another emerging challenge in exosomal therapy is that natural exosomes, also known as cell-derived exosomes, contain a multitude of biomolecules with unknown effects. Cells in different environmental conditions release exosomes with diverse cargo, complicating the predictability of cell-derived exosome therapies (262). It also makes finding the effective agent challenging. Therefore, a comprehensive review of the content of exosomes and their effect on pathologic and healthy tissues is also necessary. Additionally, although exosomes are capable of cell targeting, concerns about their intrahepatic and intrasplenic accumulation remain to be solved, as well as their relatively short half-life. These problems may be manageable if different tissue targeting strategies are used in conjunction (168). Finally, suppressing exosome secretion or function as a therapeutic strategy is far from utilization, as it requires a further in-depth understanding of the exosome's role in normal physiology (259). Accordingly, there are still serious challenges in using exosome-based therapies as a routine clinical option that should be overcome.

## 5 Conclusion

Recent findings on exosomes have provided promising prospects of using them as feasible, costly, and reliable diagnostic options in NAFLD. In addition, exosomal-based therapies, having low immunogenicity and high biocompatibility, for drug delivery as an effective treatment option or by inhibiting their actions as a treatment strategy have been associated with favorable results in NAFLD. However, despite several pre-clinical studies on exosomes in liver disorders, the passage to the clinical setting remains unsure. There are still certain challenges to using exosomal-based therapies in NAFLD. Besides, the feasibility of exosome-related treatment strategies in NAFLD will await further evaluation due to the critical role of exosomes in numerous physiological conditions. Due to the great potential of this novel theragnostic agent in NAFLD, further investigations on their safety, clinical efficacy, and application standardization are highly recommended.

## Author contributions

AT: Conceptualization, Investigation, Supervision, Validation, Visualization, Writing – original draft, Writing – review & editing. MJ: Investigation, Writing – original draft. NS-P: Visualization, Writing – review & editing. AM: Investigation, Writing – original draft.

## References

[1] Chalasani N Younossi Z Lavine JE Diehl AM Brunt EM Cusi K. The diagnosis and management of non-alcoholic fatty liver disease: practice guideline by the American Association for the Study of Liver Diseases, American College of Gastroenterology, and the American Gastroenterological Association. Hepatology. (2012) 55:2005–23. 10.1002/hep.25762 22488764

[2] Welsh JA Karpen S Vos MB. Increasing prevalence of nonalcoholic fatty liver disease among United States adolescents, 1988-1994 to 2007-2010. J Pediatr. (2013) 162:496–500.e1. 10.1016/j.jpeds.2012.08.043 23084707 PMC3649872

[3] Eguchi Y Hyogo H Ono M Mizuta T Ono N Fujimoto K. Prevalence and associated metabolic factors of nonalcoholic fatty liver disease in the general population from 2009 to 2010 in Japan: a multicenter large retrospective study. J Gastroenterol. (2012) 47:586–95. 10.1007/s00535-012-0533-z 22328022

[4] De A Duseja A. Natural history of simple steatosis or nonalcoholic fatty liver. J Clin Exp Hepatol. (2020) 10:255–62. 10.1016/j.jceh.2019.09.005 32405182 PMC7212297

[5] Loomba R Sanyal AJ. The global NAFLD epidemic. Nat Rev Gastroenterol Hepatol. (2013) 10:686–90. 10.1038/nrgastro.2013.171 24042449

[6] Friedman SL Neuschwander-Tetri BA Rinella M Sanyal AJ. Mechanisms of NAFLD development and therapeutic strategies. Nat Med. (2018) 24:908–22. 10.1038/s41591-018-0104-9 29967350 PMC6553468

[7] Rowe IA Wong VW-S Loomba R. Treatment candidacy for pharmacologic therapies for NASH. Clin Gastroenterol Hepatol. (2021) 3:5. 10.1016/j.cgh.2021.03.005 PMC8908435 33711479

[8] Muthu S Bapat A Jain R Jeyaraman N Jeyaraman M. Exosomal therapy-a new frontier in regenerative medicine. Stem Cell Invest. (2021) 8:37. 10.21037/sci-2020-037 PMC8100822 33969112

[9] Jeyaraman M Muthu S Gulati A Jeyaraman N Jain R. Mesenchymal stem cell-derived exosomes: a potential therapeutic avenue in knee osteoarthritis. Cartilage. (2021) 13(1_Suppl.):1572S−85S. 10.1177/1947603520962567 PMC8808857 33016114

[10] Théry C Witwer KW Aikawa E Alcaraz MJ Anderson JD Andriantsitohaina R. Minimal information for studies of extracellular vesicles 2018 (MISEV2018): a position statement of the International Society for Extracellular Vesicles and update of the MISEV2014 guidelines. J Extracell Vesicl. (2018) 7:1535750. 10.1080/20013078.2018.1535750 PMC6322352 30637094

[11] Cocucci E Meldolesi J. Ectosomes and exosomes: shedding the confusion between extracellular vesicles. Trends Cell Biol. (2015) 25:364–72. 10.1016/j.tcb.2015.01.004 25683921

[12] Caby M-P Lankar D Vincendeau-Scherrer C Raposo G Bonnerot C. Exosomal-like vesicles are present in human blood plasma. Int Immunol. (2005) 17:879–87. 10.1093/intimm/dxh267 15908444

[13] Pisitkun T Shen R-F Knepper MA. Identification and proteomic profiling of exosomes in human urine. Proc Nat Acad Sci USA. (2004) 101:13368–73. 10.1073/pnas.0403453101 15326289 PMC516573

[14] Michael A Bajracharya SD Yuen PS Zhou H Star RA Illei GG. Exosomes from human saliva as a source of microRNA biomarkers. Oral Dis. (2010) 16:34–8. 10.1111/j.1601-0825.2009.01604.x 19627513 PMC2844919

[15] Admyre C Johansson SM Qazi KR Filén J-J Lahesmaa R Norman M. Exosomes with immune modulatory features are present in human breast milk. J Immunol. (2007) 179:1969–78. 10.4049/jimmunol.179.3.1969 17641064

[16] Mathivanan S Fahner CJ Reid GE Simpson RJ. ExoCarta 2012: database of exosomal proteins, RNA and lipids. Nucleic Acids Res. (2012) 40:D1241–D4. 10.1093/nar/gkr828 21989406 PMC3245025

[17] Théry C Ostrowski M Segura E. Membrane vesicles as conveyors of immune responses. Nat Rev Immunol. (2009) 9:581–93. 10.1038/nri2567 19498381

[18] Daaboul GG Gagni P Benussi L Bettotti P Ciani M Cretich M. Digital detection of exosomes by interferometric imaging. Sci Rep. (2016) 6:1–10. 10.1038/srep37246 27853258 PMC5112555

[19] Pitt JM Kroemer G Zitvogel L. Extracellular vesicles: masters of intercellular communication and potential clinical interventions. J Clin Invest. (2016) 126:1139–43. 10.1172/JCI87316 27035805 PMC4811136

[20] Van Niel G d'Angelo G Raposo G. Shedding light on the cell biology of extracellular vesicles. Nat Rev Mol Cell Biol. (2018) 19:213–28. 10.1038/nrm.2017.125 29339798

[21] Xie F Zhou X Fang M Li H Su P Tu Y. Extracellular vesicles in cancer immune microenvironment and cancer immunotherapy. Adv Sci. (2019) 6:1901779. 10.1002/advs.201901779 PMC6918121 31871860

[22] Valadi H Ekström K Bossios A Sjöstrand M Lee JJ Lötvall JO. Exosome-mediated transfer of mRNAs and microRNAs is a novel mechanism of genetic exchange between cells. Nat Cell Biol. (2007) 9:654–9. 10.1038/ncb1596 17486113

[23] Kalluri R LeBleu VS. The biology, function, and biomedical applications of exosomes. Science. (2020) 367:eaau6977. 10.1126/science.aau6977 32029601 PMC7717626

[24] Krylova SV Feng D. The machinery of exosomes: biogenesis, release, and uptake. Int J Mol Sci. (2023) 24:1337. 10.3390/ijms24021337 36674857 PMC9865891

[25] Han Q-F Li W-J Hu K-S Gao J Zhai W-L Yang J-H. Exosome biogenesis: machinery, regulation, and therapeutic implications in cancer. Mol Cancer. (2022) 21:207. 10.1186/s12943-022-01671-0 36320056 PMC9623991

[26] Xie S Zhang Q Jiang L. Current knowledge on exosome biogenesis, cargo-sorting mechanism and therapeutic implications. Membranes. (2022) 12:498. 10.3390/membranes12050498 35629824 PMC9144303

[27] Hessvik NP Llorente A. Current knowledge on exosome biogenesis and release. Cell Mol Life Sci. (2018) 75:193–208. 10.1007/s00018-017-2595-9 28733901 PMC5756260

[28] Zhang Y Liu Y Liu H Tang WH. Exosomes: biogenesis, biologic function and clinical potential. Cell Biosci. (2019) 9:1–18. 10.1186/s13578-019-0282-2 30815248 PMC6377728

[29] Lau NCH Yam JWP. From exosome biogenesis to absorption: key takeaways for cancer research. Cancers. (2023) 15:1992. 10.3390/cancers15071992 37046653 PMC10093369

[30] Andreu Z Yáñez-Mó M. Tetraspanins in extracellular vesicle formation and function. Front Immunol. (2014) 5:442. 10.3389/fimmu.2014.00442 25278937 PMC4165315

[31] Larios J Mercier V Roux A Gruenberg J. ALIX-and ESCRT-III-dependent sorting of tetraspanins to exosomes. J Cell Biol. (2020) 219:4113. 10.1083/jcb.201904113 PMC7054990 32049272

[32] Kalluri R. The biology and function of exosomes in cancer. J Clin Invest. (2016) 126:1208–15. 10.1172/JCI81135 27035812 PMC4811149

[33] Pegtel DM Gould SJ. Exosomes. Annu Rev Biochem. (2019) 88:487–514. 10.1146/annurev-biochem-013118-111902 31220978

[34] Robbins PD Morelli AE. Regulation of immune responses by extracellular vesicles. Nat Rev Immunol. (2014) 14:195–208. 10.1038/nri3622 24566916 PMC4350779

[35] Becker A Thakur BK Weiss JM Kim HS Peinado H Lyden D. Extracellular vesicles in cancer: cell-to-cell mediators of metastasis. Cancer Cell. (2016) 30:836–48. 10.1016/j.ccell.2016.10.009 27960084 PMC5157696

[36] Soung YH Ford S Zhang V Chung J. Exosomes in cancer diagnostics. Cancers. (2017) 9:8. 10.3390/cancers9010008 28085080 PMC5295779

[37] Zhang Y Hu Y-W Zheng L Wang Q. Characteristics and roles of exosomes in cardiovascular disease. DNA Cell Biol. (2017) 36:202–11. 10.1089/dna.2016.3496 28112546

[38] Zhang W Zhou X Zhang H Yao Q Liu Y Dong Z. Extracellular vesicles in diagnosis and therapy of kidney diseases. Am J Physiol Renal Physiol. (2016) 311:F844–F51. 10.1152/ajprenal.00429.2016 27582107 PMC5130456

[39] Alipoor SD Mortaz E Varahram M Movassaghi M Kraneveld AD Garssen J. The potential biomarkers and immunological effects of tumor-derived exosomes in lung cancer. Front Immunol. (2018) 9:819. 10.3389/fimmu.2018.00819 29720982 PMC5915468

[40] Kanninen KM Bister N Koistinaho J Malm T. Exosomes as new diagnostic tools in CNS diseases. Biochimica et Biophysica Acta. (2016) 1862:403–10. 10.1016/j.bbadis.2015.09.020 26432482

[41] Fitts CA Ji N Li Y Tan C. Exploiting exosomes in cancer liquid biopsies and drug delivery. Adv Healthc Mater. (2019) 8:1801268. 10.1002/adhm.201801268 30663276

[42] Masyuk AI Masyuk TV LaRusso NF. Exosomes in the pathogenesis, diagnostics and therapeutics of liver diseases. J Hepatol. (2013) 59:621–5. 10.1016/j.jhep.2013.03.028 23557871 PMC3831338

[43] Yang Y Hong Y Cho E Kim GB Kim I-S. Extracellular vesicles as a platform for membrane-associated therapeutic protein delivery. J Extracell Vesicl. (2018) 7:1440131. 10.1080/20013078.2018.1440131 PMC5844050 29535849

[44] Jiang X-C Gao J-Q. Exosomes as novel bio-carriers for gene and drug delivery. Int J Pharm. (2017) 521:167–75. 10.1016/j.ijpharm.2017.02.038 28216464

[45] Zhang B Yin Y Lai RC Tan SS Choo ABH Lim SK. Mesenchymal stem cells secrete immunologically active exosomes. Stem Cells Dev. (2014) 23:1233–44. 10.1089/scd.2013.0479 24367916

[46] Lee C Mitsialis SA Aslam M Vitali SH Vergadi E Konstantinou G. Exosomes mediate the cytoprotective action of mesenchymal stromal cells on hypoxia-induced pulmonary hypertension. Circulation. (2012) 126:2601–11. 10.1161/CIRCULATIONAHA.112.114173 23114789 PMC3979353

[47] Kanazawa H Fujimoto Y Teratani T Iwasaki J Kasahara N Negishi K. Bone marrow-derived mesenchymal stem cells ameliorate hepatic ischemia reperfusion injury in a rat model. PLoS ONE. (2011) 6:e19195. 10.1371/journal.pone.0019195 21559442 PMC3084802

[48] Lai RC Arslan F Lee MM Sze NSK Choo A Chen TS. Exosome secreted by MSC reduces myocardial ischemia/reperfusion injury. Stem Cell Res. (2010) 4:214–22. 10.1016/j.scr.2009.12.003 20138817

[49] Salomon C Ryan J Sobrevia L Kobayashi M Ashman K Mitchell M. Exosomal signaling during hypoxia mediates microvascular endothelial cell migration and vasculogenesis. PLoS ONE. (2013) 8:e68451. 10.1371/journal.pone.0068451 23861904 PMC3704530

[50] Chen J Liu Z Hong MM Zhang H Chen C Xiao M. Proangiogenic compositions of microvesicles derived from human umbilical cord mesenchymal stem cells. PLoS ONE. (2014) 9:e115316. 10.1371/journal.pone.0115316 25514634 PMC4267846

[51] Rani S Ryan AE Griffin MD Ritter T. Mesenchymal stem cell-derived extracellular vesicles: toward cell-free therapeutic applications. Mol Ther. (2015) 23:812–23. 10.1038/mt.2015.44 25868399 PMC4427881

[52] Fang YL Chen H Wang CL Liang L. Pathogenesis of non-alcoholic fatty liver disease in children and adolescence: From “two hit theory” to “multiple hit model”. World J Gastroenterol. (2018) 24:2974–83. 10.3748/wjg.v24.i27.2974 30038464 PMC6054950

[53] Schwimmer JB Behling C Newbury R Deutsch R Nievergelt C Schork NJ. Histopathology of pediatric nonalcoholic fatty liver disease. Hepatology. (2005) 42:641–9. 10.1002/hep.20842 16116629

[54] Ratziu V Bellentani S Cortez-Pinto H Day C Marchesini G. A position statement on NAFLD/NASH based on the EASL 2009 special conference. J Hepatol. (2010) 53:372–84. 10.1016/j.jhep.2010.04.008 20494470

[55] Berardis S Sokal E. Pediatric non-alcoholic fatty liver disease: an increasing public health issue. Eur J Pediatr. (2014) 173:131–9. 10.1007/s00431-013-2157-6 24068459 PMC3929043

[56] Alisi A Cianfarani S Manco M Agostoni C Nobili V. Non-alcoholic fatty liver disease and metabolic syndrome in adolescents: pathogenetic role of genetic background and intrauterine environment. Ann Med. (2012) 44:29–40. 10.3109/07853890.2010.547869 21355790

[57] Ayonrinde OT Olynyk JK Marsh JA Beilin LJ Mori TA Oddy WH. Childhood adiposity trajectories and risk of nonalcoholic fatty liver disease in adolescents. J Gastroenterol Hepatol. (2015) 30:163–71. 10.1111/jgh.12666 24989077

[58] Cohen JC Horton JD Hobbs HH. Human fatty liver disease: old questions and new insights. Science. (2011) 332:1519–23. 10.1126/science.1204265 21700865 PMC3229276

[59] Lomonaco R Ortiz-Lopez C Orsak B Webb A Hardies J Darland C. Effect of adipose tissue insulin resistance on metabolic parameters and liver histology in obese patients with nonalcoholic fatty liver disease. Hepatology. (2012) 55:1389–97. 10.1002/hep.25539 22183689

[60] Donnelly KL Smith CI Schwarzenberg SJ Jessurun J Boldt MD Parks EJ. Sources of fatty acids stored in liver and secreted via lipoproteins in patients with nonalcoholic fatty liver disease. J Clin Invest. (2005) 115:1343–51. 10.1172/JCI200523621 15864352 PMC1087172

[61] Han J Kaufman RJ. The role of ER stress in lipid metabolism and lipotoxicity. J Lipid Res. (2016) 57:1329–38. 10.1194/jlr.R067595 27146479 PMC4959874

[62] Puri P Mirshahi F Cheung O Natarajan R Maher JW Kellum JM. Activation and dysregulation of the unfolded protein response in nonalcoholic fatty liver disease. Gastroenterology. (2008) 134:568–76. 10.1053/j.gastro.2007.10.039 18082745

[63] Szabo G Petrasek J. Inflammasome activation and function in liver disease. Nat Rev Gastroenterol Hepatol. (2015) 12:387–400. 10.1038/nrgastro.2015.94 26055245

[64] Guy CD Suzuki A Zdanowicz M Abdelmalek MF Burchette J Unalp A. Hedgehog pathway activation parallels histologic severity of injury and fibrosis in human nonalcoholic fatty liver disease. Hepatology. (2012) 55:1711–21. 10.1002/hep.25559 22213086 PMC3499103

[65] Loomba R Abraham M Unalp A Wilson L Lavine J Doo E. Association between diabetes, family history of diabetes, and risk of nonalcoholic steatohepatitis and fibrosis. Hepatology. (2012) 56:943–51. 10.1002/hep.25772 22505194 PMC3407289

[66] Marra F Bertolani C. Adipokines in liver diseases. Hepatology. (2009) 50:957–69. 10.1002/hep.23046 19585655

[67] Abdelmalek MF Lazo M Horska A Bonekamp S Lipkin EW Balasubramanyam A. Higher dietary fructose is associated with impaired hepatic adenosine triphosphate homeostasis in obese individuals with type 2 diabetes. Hepatology. (2012) 56:952–60. 10.1002/hep.25741 22467259 PMC3406258

[68] Lanaspa MA Sanchez-Lozada LG Choi YJ Cicerchi C Kanbay M Roncal-Jimenez CA. Uric acid induces hepatic steatosis by generation of mitochondrial oxidative stress: potential role in fructose-dependent and -independent fatty liver. J Biol Chem. (2012) 287:40732–44. 10.1074/jbc.M112.399899 23035112 PMC3504786

[69] Henao-Mejia J Elinav E Jin C Hao L Mehal WZ Strowig T. Inflammasome-mediated dysbiosis regulates progression of NAFLD and obesity. Nature. (2012) 482:179–85. 10.1038/nature10809 22297845 PMC3276682

[70] Tsuchida T Friedman SL. Mechanisms of hepatic stellate cell activation. Nat Rev Gastroenterol Hepatol. (2017) 14:397–411. 10.1038/nrgastro.2017.38 28487545

[71] Sato K Meng F Glaser S Alpini G. Exosomes in liver pathology. J Hepatol. (2016) 65:213–21. 10.1016/j.jhep.2016.03.004 26988731 PMC4912847

[72] Povero D Eguchi A Li H Johnson CD Papouchado BG Wree A. Circulating extracellular vesicles with specific proteome and liver microRNAs are potential biomarkers for liver injury in experimental fatty liver disease. PLoS ONE. (2014) 9:e113651. 10.1371/journal.pone.0113651 25470250 PMC4254757

[73] Yao ZY Chen WB Shao SS Ma SZ Yang CB Li MZ. Role of exosome-associated microRNA in diagnostic and therapeutic applications to metabolic disorders. J Zhejiang Univ Sci B. (2018) 19:183–98. 10.1631/jzus.B1600490 29504312 PMC5854634

[74] Sato K Kennedy L Liangpunsakul S Kusumanchi P Yang Z Meng F. Intercellular communication between hepatic cells in liver diseases. Int J Mol Sci. (2019) 20:92180. 10.3390/ijms20092180 PMC6540342 31052525

[75] European Association for the Study of the L, European Association for the Study of D, European Association for the Study of O. EASL-EASD-EASO Clinical Practice Guidelines for the management of non-alcoholic fatty liver disease. Diabetologia. (2016) 59:1121–40. 10.1007/s00125-016-3902-y 27053230

[76] Ban LA Shackel NA McLennan SV. Extracellular vesicles: a new frontier in biomarker discovery for non-alcoholic fatty liver disease. Int J Mol Sci. (2016) 17:376. 10.3390/ijms17030376 26985892 PMC4813235

[77] Kakazu E Mauer AS Yin M Malhi H. Hepatocytes release ceramide-enriched pro-inflammatory extracellular vesicles in an IRE1α-dependent manner. J Lipid Res. (2016) 57:233–45. 10.1194/jlr.M063412 26621917 PMC4727419

[78] Schuster S Cabrera D Arrese M Feldstein AE. Triggering and resolution of inflammation in NASH. Nat Rev Gastroenterol Hepatol. (2018) 15:349–64. 10.1038/s41575-018-0009-6 29740166

[79] Li Y Luan Y Li J Song H Li Y Qi H. Exosomal miR-199a-5p promotes hepatic lipid accumulation by modulating MST1 expression and fatty acid metabolism. Hepatol Int. (2020) 14:1057–74. 10.1007/s12072-020-10096-0 33037981

[80] Xu Y Zhu Y Hu S Pan X Bawa FC Wang HH. Hepatocyte miR-34a is a key regulator in the development and progression of non-alcoholic fatty liver disease. Mol Metab. (2021) 51:101244. 10.1016/j.molmet.2021.101244 33930596 PMC8141777

[81] Arrese M Cabrera D Kalergis AM Feldstein AE. Innate immunity and inflammation in NAFLD/NASH. Dig Dis Sci. (2016) 61:1294–303. 10.1007/s10620-016-4049-x 26841783 PMC4948286

[82] Hirsova P Ibrahim SH Krishnan A Verma VK Bronk SF Werneburg NW. Lipid-induced signaling causes release of inflammatory extracellular vesicles from hepatocytes. Gastroenterology. (2016) 150:956–67. 10.1053/j.gastro.2015.12.037 26764184 PMC4808464

[83] Ibrahim SH Hirsova P Tomita K Bronk SF Werneburg NW Harrison SA. Mixed lineage kinase 3 mediates release of C-X-C motif ligand 10-bearing chemotactic extracellular vesicles from lipotoxic hepatocytes. Hepatology. (2016) 63:731–44. 10.1002/hep.28252 26406121 PMC4764421

[84] Nakao Y Fukushima M Mauer AS Liao CY Ferris A Dasgupta D. A comparative proteomic analysis of extracellular vesicles associated with lipotoxicity. Front Cell Dev Biol. (2021) 9:735001. 10.3389/fcell.2021.735001 34805145 PMC8600144

[85] Dasgupta D Nakao Y Mauer AS Thompson JM Sehrawat TS Liao CY. IRE1A stimulates hepatocyte-derived extracellular vesicles that promote inflammation in mice with steatohepatitis. Gastroenterology. (2020) 159:1487–503.e17. 10.1053/j.gastro.2020.06.031 32574624 PMC7666601

[86] Vonderlin J Chavakis T Sieweke M Tacke F. The multifaceted roles of macrophages in NAFLD pathogenesis. Cell Mol Gastroenterol Hepatol. (2023) 15:1311–24. 10.1016/j.jcmgh.2023.03.002 36907380 PMC10148157

[87] Wang C Ma C Gong L Guo Y Fu K Zhang Y. Macrophage polarization and its role in liver disease. Front Immunol. (2021) 12:803037. 10.3389/fimmu.2021.803037 34970275 PMC8712501

[88] Shen M Shen Y Fan X Men R Ye T Yang L. Roles of macrophages and exosomes in liver diseases. Front Med. (2020) 7:583691. 10.3389/fmed.2020.583691 PMC7542243 33072790

[89] Lu W Bai L Chen Y. The role of macrophage-derived exosomes in liver diseases. Infect Dis Immunity. (2022) 2:34. 10.1097/ID9.0000000000000034

[90] Liu XL Cao HX Fan JG. MicroRNAs as biomarkers and regulators of nonalcoholic fatty liver disease. J Dig Dis. (2016) 17:708–15. 10.1111/1751-2980.12408 27628945

[91] Liu X-L Pan Q Cao H-X Xin F-Z Zhao Z-H Yang R-X. Lipotoxic hepatocyte-derived exosomal MicroRNA 192-5p activates macrophages through Rictor/Akt/Forkhead box transcription factor O1 signaling in nonalcoholic fatty liver disease. Hepatology. (2020) 72:454–69. 10.1002/hep.31050 31782176 PMC10465073

[92] Zhao Z Zhong L Li P He K Qiu C Zhao L. Cholesterol impairs hepatocyte lysosomal function causing M1 polarization of macrophages via exosomal miR-122-5p. Exp Cell Res. (2020) 387:111738. 10.1016/j.yexcr.2019.111738 31759057

[93] Cheng D Chai J Wang H Fu L Peng S Ni X. Hepatic macrophages: key players in the development and progression of liver fibrosis. Liver Int. (2021) 41:2279–94. 10.1111/liv.14940 33966318

[94] Wan Z Yang X Liu X Sun Y Yu P Xu F. M2 macrophage-derived exosomal microRNA-411-5p impedes the activation of hepatic stellate cells by targeting CAMSAP1 in NASH model. iScience. (2022) 25:104597. 10.1016/j.isci.2022.104597 35789846 PMC9249826

[95] Sakai Y Chen G Ni Y Zhuge F Xu L Nagata N. DPP-4 inhibition with anagliptin reduces lipotoxicity-induced insulin resistance and steatohepatitis in male mice. Endocrinology. (2020) 161:bqaa139. 10.1210/endocr/bqaa139 32790863

[96] Su SB Qin SY Xian XL Huang FF Huang QL Zhang Di HJ. Interleukin-22 regulating Kupffer cell polarization through STAT3/Erk/Akt crosstalk pathways to extenuate liver fibrosis. Life Sci. (2021) 264:118677. 10.1016/j.lfs.2020.118677 33129875

[97] Yang Y Wu XQ Li WX Huang HM Li HD Pan XY. PSTPIP2 connects DNA methylation to macrophage polarization in CCL4-induced mouse model of hepatic fibrosis. Oncogene. (2018) 37:6119–35. 10.1038/s41388-018-0383-0 29993036

[98] Chen T Liu R Niu Y Mo H Wang H Lu Y. HIF-1α-activated long non-coding RNA KDM4A-AS1 promotes hepatocellular carcinoma progression via the miR-411-5p/KPNA2/AKT pathway. Cell Death Dis. (2021) 12:1152. 10.1038/s41419-021-04449-2 34903711 PMC8668937

[99] Duan WJ Li YF Liu FL Deng J Wu YP Yuan WL. A SIRT3/AMPK/autophagy network orchestrates the protective effects of trans-resveratrol in stressed peritoneal macrophages and RAW 2647 macrophages. Free Rad Biol Med. (2016) 95:230–42. 10.1016/j.freeradbiomed.2016.03.022 27021965

[100] Yang L Liu S He Y Gan L Ni Q Dai A. Exosomes regulate SIRT3-related autophagy by delivering miR-421 to regulate macrophage polarization and participate in OSA-related NAFLD. J Transl Med. (2024) 22:475. 10.1186/s12967-024-05283-8 38764033 PMC11103849

[101] Shen Y Malik SA Amir M Kumar P Cingolani F Wen J. Decreased hepatocyte autophagy leads to synergistic IL-1β and TNF mouse liver injury and inflammation. Hepatology. (2020) 72:595–608. 10.1002/hep.31209 32108953 PMC8114460

[102] Balaphas A Meyer J Sadoul K Fontana P Morel P Gonelle-Gispert C. Platelets and platelet-derived extracellular vesicles in liver physiology and disease. Hepatol Commun. (2019) 3:855–66. 10.1002/hep4.1358 31304449 PMC6601322

[103] Chauhan A Adams DH Watson SP Lalor PF. Platelets: no longer bystanders in liver disease. Hepatology. (2016) 64:1774–84. 10.1002/hep.28526 26934463 PMC5082495

[104] Lee Y-S Kim SY Ko E Lee J-H Yi H-S Yoo YJ. Exosomes derived from palmitic acid-treated hepatocytes induce fibrotic activation of hepatic stellate cells. Sci Rep. (2017) 7:3710. 10.1038/s41598-017-03389-2 28623272 PMC5473841

[105] Wang W Li F Lai X Liu H Wu S Han Y. Exosomes secreted by palmitic acid-treated hepatocytes promote LX-2 cell activation by transferring miRNA-107. Cell Death Discov. (2021) 7:174. 10.1038/s41420-021-00536-7 34234100 PMC8263701

[106] Qi Y Ren S Ye J Tian Y Wang G Zhang S. Infection microenvironment-activated core-shell nanoassemblies for photothermal/chemodynamic synergistic wound therapy and multimodal imaging. Acta Biomater. (2022) 143:445–58. 10.1016/j.actbio.2022.02.034 35235864

[107] Hou X Yin S Ren R Liu S Yong L Liu Y. Myeloid-cell-specific IL-6 signaling promotes MicroRNA-223-enriched exosome production to attenuate NAFLD-associated fibrosis. Hepatology. (2021) 74:116–32. 10.1002/hep.31658 33236445 PMC8141545

[108] Afrisham R Sadegh-Nejadi S Meshkani R Emamgholipour S Paknejad M. Effect of circulating exosomes derived from normal-weight and obese women on gluconeogenesis, glycogenesis, lipogenesis and secretion of FGF21 and fetuin A in HepG2 cells. Diabetol Metab Syndr. (2020) 12:32. 10.1186/s13098-020-00540-4 32322309 PMC7161281

[109] Koeck ES Iordanskaia T Sevilla S Ferrante SC Hubal MJ Freishtat RJ. Adipocyte exosomes induce transforming growth factor beta pathway dysregulation in hepatocytes: a novel paradigm for obesity-related liver disease. J Surg Res. (2014) 192:268–75. 10.1016/j.jss.2014.06.050 25086727

[110] Baranova A Maltseva D Tonevitsky A. Adipose may actively delay progression of NAFLD by releasing tumor-suppressing, anti-fibrotic miR-122 into circulation. Obes Rev. (2019) 20:108–18. 10.1111/obr.12765 30248223

[111] Ogur HU Kapukaya R Çiloglu O Külahci Ö Tekbaş VT Yörükoglu A. Treatment of open wounds secondary to trauma using polyurethane foams with boric acid particles. Ulusal travma ve acil cerrahi dergisi. (2021) 27:624–30. 10.14744/tjtes.2020.38613 34710221

[112] Yan C Tian X Li J Liu D Ye D Xie Z. A high-fat diet attenuates AMPK α1 in adipocytes to induce exosome shedding and nonalcoholic fatty liver development *in vivo*. Diabetes. (2021) 70:577–88. 10.2337/db20-0146 33262120 PMC7881856

[113] Zhao J Hu L Gui W Xiao L Wang W Xia J. Hepatocyte TGF-β signaling inhibiting WAT browning to promote NAFLD and obesity is associated with Let-7b-5p. Hepatol Commun. (2022) 6:1301–21. 10.1002/hep4.1892 35018737 PMC9134819

[114] Fuchs A Samovski D Smith GI Cifarelli V Farabi SS Yoshino J. Associations among adipose tissue immunology, inflammation, exosomes and insulin sensitivity in people with obesity and nonalcoholic fatty liver disease. Gastroenterology. (2021) 161:968–81.e12. 10.1053/j.gastro.2021.05.008 34004161 PMC8900214

[115] Sakurai Y Kubota N Yamauchi T Kadowaki T. Role of insulin resistance in MAFLD. Int J Mol Sci. (2021) 22:4156. 10.3390/ijms22084156 33923817 PMC8072900

[116] Zhao H Shang Q Pan Z Bai Y Li Z Zhang H. Exosomes from adipose-derived stem cells attenuate adipose inflammation and obesity through polarizing M2 macrophages and beiging in white adipose tissue. Diabetes. (2018) 67:235–47. 10.2337/db17-0356 29133512

[117] Chen Y Sun H Bai Y Zhi F. Gut dysbiosis-derived exosomes trigger hepatic steatosis by transiting HMGB1 from intestinal to liver in mice. Biochem Biophys Res Commun. (2019) 509:767–72. 10.1016/j.bbrc.2018.12.180 30616887

[118] Ma S Shao S Yang C Yao Z Gao L Chen W. preliminary study: proteomic analysis of exosomes derived from thyroid-stimulating hormone-stimulated HepG2 cells. J Endocrinol Invest. (2020) 43:1229–38. 10.1007/s40618-020-01210-y 32166700

[119] Zheng D Huo M Li B Wang W Piao H Wang Y. The role of exosomes and exosomal MicroRNA in cardiovascular disease. Front Cell Develop Biol. (2021) 8:616161. 10.3389/fcell.2020.616161 PMC7835482 33511124

[120] Zhang JW Pan HT. microRNA profiles of serum exosomes derived from children with nonalcoholic fatty liver. Genes Genomics. (2022) 44:879–88. 10.1007/s13258-021-01150-8 34390467

[121] Zhou X Huang K Jia J Ni Y Yuan J Liang X. Exosomal miRNAs profile in children's nonalcoholic fatty liver disease and the correlation with transaminase and uric acid. Ann Nutr Metab. (2020) 76:44–53. 10.1159/000506665 32172249

[122] Lewis AP Jopling CL. Regulation and biological function of the liver-specific miR-122. Biochem Soc Trans. (2010) 38:1553–7. 10.1042/BST0381553 21118125

[123] Pirola CJ Fernández Gianotti T Castaño GO Mallardi P San Martino J Mora Gonzalez Lopez Ledesma M. Circulating microRNA signature in non-alcoholic fatty liver disease: from serum non-coding RNAs to liver histology and disease pathogenesis. Gut. (2015) 64:800–12. 10.1136/gutjnl-2014-306996 24973316 PMC4277726

[124] Benhamouche-Trouillet S Postic C. Emerging role of miR-21 in non-alcoholic fatty liver disease. Gut. (2016) 65:1781–3. 10.1136/gutjnl-2015-310044 27436271

[125] Loyer X Paradis V Hénique C Vion AC Colnot N Guerin CL. Liver microRNA-21 is overexpressed in non-alcoholic steatohepatitis and contributes to the disease in experimental models by inhibiting PPARα expression. Gut. (2016) 65:1882–94. 10.1136/gutjnl-2014-308883 26338827 PMC5099209

[126] Ahn J Lee H Jung CH Ha T. Lycopene inhibits hepatic steatosis via microRNA-21-induced downregulation of fatty acid-binding protein 7 in mice fed a high-fat diet. Mol Nutr Food Res. (2012) 56:1665–74. 10.1002/mnfr.201200182 22968990

[127] Sun C Huang F Liu X Xiao X Yang M Hu G. miR-21 regulates triglyceride and cholesterol metabolism in non-alcoholic fatty liver disease by targeting HMGCR. Int J Mol Med. (2015) 35:847–53. 10.3892/ijmm.2015.2076 25605429

[128] Wu H Ng R Chen X Steer CJ Song G. MicroRNA-21 is a potential link between non-alcoholic fatty liver disease and hepatocellular carcinoma via modulation of the HBP1-p53-Srebp1c pathway. Gut. (2016) 65:1850–60. 10.1136/gutjnl-2014-308430 26282675 PMC4882277

[129] Dattaroy D Pourhoseini S Das S Alhasson F Seth RK Nagarkatti M. Micro-RNA 21 inhibition of SMAD7 enhances fibrogenesis via leptin-mediated NADPH oxidase in experimental and human nonalcoholic steatohepatitis. Am J Physiol Gastrointest Liver Physiol. (2015) 308:G298–312. 10.1152/ajpgi.00346.2014 25501551 PMC4329476

[130] Caviglia JM Yan J Jang MK Gwak GY Affo S Yu L. MicroRNA-21 and Dicer are dispensable for hepatic stellate cell activation and the development of liver fibrosis. Hepatology. (2018) 67:2414–29. 10.1002/hep.29627 29091291 PMC5930143

[131] Long JK Dai W Zheng YW Zhao SP. miR-122 promotes hepatic lipogenesis via inhibiting the LKB1/AMPK pathway by targeting Sirt1 in non-alcoholic fatty liver disease. Mol Med. (2019) 25:26. 10.1186/s10020-019-0085-2 31195981 PMC6567918

[132] Ding RB Bao J Deng CX. Emerging roles of SIRT1 in fatty liver diseases. Int J Biol Sci. (2017) 13:852–67. 10.7150/ijbs.19370 28808418 PMC5555103

[133] Zhang M Sun W Zhou M Tang Y. MicroRNA-27a regulates hepatic lipid metabolism and alleviates NAFLD via repressing FAS and SCD1. Sci Rep. (2017) 7:14493. 10.1038/s41598-017-15141-x 29101357 PMC5670231

[134] Luo X Xu ZX Wu JC Luo SZ Xu MY. Hepatocyte-derived exosomal miR-27a activateshepatic stellate cells through the inhibitionof PINK1-mediated mitophagy in MAFLD. Mol Ther Nucleic Acids. (2021) 26:1241–54. 10.1016/j.omtn.2021.10.022 34853724 PMC8607138

[135] Shen M Pan H Ke J Zhao F. NF-κB-upregulated miR-155-5p promotes hepatocyte mitochondrial dysfunction to accelerate the development of nonalcoholic fatty liver disease through downregulation of STC1. J Biochem Mol Toxicol. (2022) 36:e23025. 10.1002/jbt.23025 35603999

[136] López-Pastor AR Infante-Menéndez J Escribano Ó Gómez-Hernández A. miRNA dysregulation in the development of non-alcoholic fatty liver disease and the related disorders type 2 diabetes mellitus and cardiovascular disease. Front Med. (2020) 7:527059. 10.3389/fmed.2020.527059 PMC7546803 33102495

[137] González-López P Ares-Carral C López-Pastor AR Infante-Menéndez J González Illaness T Vega de Ceniga M. Implication of miR-155-5p and miR-143-3p in the vascular insulin resistance and instability of human and experimental atherosclerotic plaque. Int J Mol Sci. (2022) 23:1810253. 10.3390/ijms231810253 PMC9499612 36142173

[138] Ding J Li M Wan X Jin X Chen S Yu C. Effect of miR-34a in regulating steatosis by targeting PPARα expression in nonalcoholic fatty liver disease. Sci Rep. (2015) 5:13729. 10.1038/srep13729 26330104 PMC4557122

[139] Lin Y Ding D Huang Q Liu Q Lu H Lu Y. Downregulation of miR-192 causes hepatic steatosis and lipid accumulation by inducing SREBF1: Novel mechanism for bisphenol A-triggered non-alcoholic fatty liver disease. Biochim Biophys Acta Mol Cell Biol Lipids. (2017) 1862:869–82. 10.1016/j.bbalip.2017.05.001 28483554

[140] Du X Yang Y Xu C Peng Z Zhang M Lei L. Upregulation of miR-181a impairs hepatic glucose and lipid homeostasis. Oncotarget. (2017) 8:91362–78. 10.18632/oncotarget.20523 29207650 PMC5710930

[141] Yang YL Wang PW Wang FS Lin HY Huang YH. miR-29a Modulates GSK3β/SIRT1-linked mitochondrial proteostatic stress to ameliorate mouse non-alcoholic steatohepatitis. Int J Mol Sci. (2020) 21:186884. 10.3390/ijms21186884 PMC7555728 32961796

[142] Gim JA Bang SM Lee YS Lee Y Yim SY Jung YK. Evaluation of the severity of nonalcoholic fatty liver disease through analysis of serum exosomal miRNA expression. PLoS ONE. (2021) 16:e0255822. 10.1371/journal.pone.0255822 34358264 PMC8345824

[143] Nguyen HQ Lee D Kim Y Bang G Cho K Lee YS. Label-free quantitative proteomic analysis of serum extracellular vesicles differentiating patients of alcoholic and nonalcoholic fatty liver diseases. J Proteomics. (2021) 245:104278. 10.1016/j.jprot.2021.104278 34089894 PMC8277700

[144] Scavo MP Depalo N Rizzi F Carrieri L Serino G Franco I. Exosomal FZD-7 expression is modulated by different lifestyle interventions in patients with NAFLD. Nutrients. (2022) 14:61133. 10.3390/nu14061133 PMC8950750 35334792

[145] Sung S Kim J Jung Y. Liver-derived exosomes and their implications in liver pathobiology. Int J Mol Sci. (2018) 19:123715. 10.3390/ijms19123715 PMC6320937 30469540

[146] Sumida Y Yoneda M. Current and future pharmacological therapies for NAFLD/NASH. J Gastroenterol. (2018) 53:362–76. 10.1007/s00535-017-1415-1 29247356 PMC5847174

[147] Chen J Vitetta L. Gut microbiota metabolites in NAFLD pathogenesis and therapeutic implications. Int J Mol Sci. (2020) 21:155214. 10.3390/ijms21155214 PMC7432372 32717871

[148] Rezaie J Feghhi M Etemadi T. A review on exosomes application in clinical trials: perspective, questions, and challenges. Cell Commun Signal. (2022) 20:145. 10.1186/s12964-022-00959-4 36123730 PMC9483361

[149] Phinney DG Pittenger MF. Concise review: MSC-derived exosomes for cell-free therapy. Stem Cells. (2017) 35:851–8. 10.1002/stem.2575 28294454

[150] Ibrahim AG Cheng K Marbán E. Exosomes as critical agents of cardiac regeneration triggered by cell therapy. Stem Cell Reports. (2014) 2:606–19. 10.1016/j.stemcr.2014.04.006 24936449 PMC4050492

[151] Blans K Hansen MS Sørensen LV Hvam ML Howard KA Möller A. Pellet-free isolation of human and bovine milk extracellular vesicles by size-exclusion chromatography. J Extracell Vesicl. (2017) 6:1294340. 10.1080/20013078.2017.1294340 PMC5373680 28386391

[152] Clayton A Boilard E Buzas EI Cheng L Falcón-Perez JM Gardiner C. Considerations towards a roadmap for collection, handling and storage of blood extracellular vesicles. J Extracell Vesicl. (2019) 8:1647027. 10.1080/20013078.2019.1647027 PMC6711123 31489143

[153] Gallo A Tandon M Alevizos I Illei GG. The majority of microRNAs detectable in serum and saliva is concentrated in exosomes. PLoS ONE. (2012) 7:e30679. 10.1371/journal.pone.0030679 22427800 PMC3302865

[154] Iwai K Minamisawa T Suga K Yajima Y Shiba K. Isolation of human salivary extracellular vesicles by iodixanol density gradient ultracentrifugation and their characterizations. J Extracell Vesicl. (2016) 5:30829. 10.3402/jev.v5.30829 PMC4871899 27193612

[155] Simpson RJ Lim JW Moritz RL Mathivanan S. Exosomes: proteomic insights and diagnostic potential. Expert Rev Proteom. (2009) 6:267–83. 10.1586/epr.09.17 19489699

[156] Srinivasan S Duval MX Kaimal V Cuff C Clarke SH. Assessment of methods for serum extracellular vesicle small RNA sequencing to support biomarker development. J Extracell Vesicl. (2019) 8:1684425. 10.1080/20013078.2019.1684425 PMC6844434 31741724

[157] Taylor DD Gercel-Taylor C. MicroRNA signatures of tumor-derived exosomes as diagnostic biomarkers of ovarian cancer. Gynecol Oncol. (2008) 110:13–21. 10.1016/j.ygyno.2008.04.033 18589210

[158] Zonneveld MI Brisson AR van Herwijnen MJ Tan S van de Lest CH Redegeld FA. Recovery of extracellular vesicles from human breast milk is influenced by sample collection and vesicle isolation procedures. J Extracell Vesicl. (2014) 3:24215. 10.3402/jev.v3.24215 PMC4139932 25206958

[159] Yeo RW Lai RC Zhang B Tan SS Yin Y Teh BJ. Mesenchymal stem cell: an efficient mass producer of exosomes for drug delivery. Adv Drug Deliv Rev. (2013) 65:336–41. 10.1016/j.addr.2012.07.001 22780955

[160] Kwon SG Kwon YW Lee TW Park GT Kim JH. Recent advances in stem cell therapeutics and tissue engineering strategies. Biomater Res. (2018) 22:36. 10.1186/s40824-018-0148-4 30598836 PMC6299977

[161] Lai RC Yeo RW Lim SK. Mesenchymal stem cell exosomes. Semin Cell Dev Biol. (2015) 40:82–8. 10.1016/j.semcdb.2015.03.001 25765629

[162] Han C Sun X Liu L Jiang H Shen Y Xu X. Exosomes and their therapeutic potentials of stem cells. Stem Cells Int. (2016) 2016:7653489. 10.1155/2016/7653489 26770213 PMC4684885

[163] Barbash IM Chouraqui P Baron J Feinberg MS Etzion S Tessone A. Systemic delivery of bone marrow-derived mesenchymal stem cells to the infarcted myocardium: feasibility, cell migration, and body distribution. Circulation. (2003) 108:863–8. 10.1161/01.CIR.0000084828.50310.6A 12900340

[164] Braid LR Wood CA Wiese DM Ford BN. Intramuscular administration potentiates extended dwell time of mesenchymal stromal cells compared to other routes. Cytotherapy. (2018) 20:232–44. 10.1016/j.jcyt.2017.09.013 29167063

[165] Jung JW Kwon M Choi JC Shin JW Park IW Choi BW. Familial occurrence of pulmonary embolism after intravenous, adipose tissue-derived stem cell therapy. Yonsei Med J. (2013) 54:1293–6. 10.3349/ymj.2013.54.5.1293 23918585 PMC3743204

[166] Yong KW Wan Safwani WK Xu F Wan Abas WA Choi JR Pingguan-Murphy B. Cryopreservation of human mesenchymal stem cells for clinical applications: current methods and challenges. Biopreserv Biobank. (2015) 13:231–9. 10.1089/bio.2014.0104 26280501

[167] Keerthikumar S Chisanga D Ariyaratne D Al Saffar H Anand S Zhao K. ExoCarta: a web-based compendium of exosomal cargo. J Mol Biol. (2016) 428:688–92. 10.1016/j.jmb.2015.09.019 26434508 PMC4783248

[168] Wu R Fan X Wang Y Shen M Zheng Y Zhao S. Mesenchymal stem cell-derived extracellular vesicles in liver immunity and therapy. Front Immunol. (2022) 13:833878. 10.3389/fimmu.2022.833878 35309311 PMC8930843

[169] Zhang Z Lin H Shi M Xu R Fu J Lv J. Human umbilical cord mesenchymal stem cells improve liver function and ascites in decompensated liver cirrhosis patients. J Gastroenterol Hepatol. (2012) 27(Suppl.2):112–20. 10.1111/j.1440-1746.2011.07024.x 22320928

[170] Chai NL Zhang XB Chen SW Fan KX Linghu EQ. Umbilical cord-derived mesenchymal stem cells alleviate liver fibrosis in rats. World J Gastroenterol. (2016) 22:6036–48. 10.3748/wjg.v22.i26.6036 27468195 PMC4948270

[171] Shi M Zhang Z Xu R Lin H Fu J Zou Z. Human mesenchymal stem cell transfusion is safe and improves liver function in acute-on-chronic liver failure patients. Stem Cells Transl Med. (2012) 1:725–31. 10.5966/sctm.2012-0034 23197664 PMC3659658

[172] Yan W Li D Chen T Tian G Zhou P Ju X. Umbilical cord MSCs reverse D-galactose-induced hepatic mitochondrial dysfunction via activation of Nrf2/HO-1 pathway. Biol Pharm Bull. (2017) 40:1174–82. 10.1248/bpb.b16-00777 28502921

[173] Yun JW Ahn JH Kwon E Kim SH Kim H Jang JJ. Human umbilical cord-derived mesenchymal stem cells in acute liver injury: Hepatoprotective efficacy, subchronic toxicity, tumorigenicity, and biodistribution. Regul Toxicol Pharmacol. (2016) 81:437–47. 10.1016/j.yrtph.2016.09.029 27693706

[174] Fan J Tang X Wang Q Zhang Z Wu S Li W. Mesenchymal stem cells alleviate experimental autoimmune cholangitis through immunosuppression and cytoprotective function mediated by galectin-9. Stem Cell Res Ther. (2018) 9:237. 10.1186/s13287-018-0979-x 30223894 PMC6142687

[175] Li B Cheng Y Yu S Zang L Yin Y Liu J. Human umbilical cord-derived mesenchymal stem cell therapy ameliorates nonalcoholic fatty liver disease in obese type 2 diabetic mice. Stem Cells Int. (2019) 2019:8628027. 10.1155/2019/8628027 31781248 PMC6875176

[176] Cheng L Yu P Li F Jiang X Jiao X Shen Y. Human umbilical cord-derived mesenchymal stem cell-exosomal miR-627-5p ameliorates non-alcoholic fatty liver disease by repressing FTO expression. Hum Cell. (2021) 34:1697–708. 10.1007/s13577-021-00593-1 34410623

[177] Schuhladen K Stich L Schmidt J Steinkasserer A Boccaccini AR Zinser E. Cu, Zn doped borate bioactive glasses: antibacterial efficacy and dose-dependent *in vitro* modulation of murine dendritic cells. Biomater Sci. (2020) 8:2143–55. 10.1039/C9BM01691K 32248211

[178] Mizuno TM. Fat mass and obesity associated (FTO) gene and hepatic glucose and lipid metabolism. Nutrients. (2018) 10:166. 10.20944/preprints201810.0166.v1 30388740 PMC6266206

[179] Kang Y Song Y Luo Y Song J Li C Yang S. Exosomes derived from human umbilical cord mesenchymal stem cells ameliorate experimental non-alcoholic steatohepatitis via Nrf2/NQO-1 pathway. Free Radic Biol Med. (2022) 192:25–36. 10.1016/j.freeradbiomed.2022.08.037 36096356

[180] Ross D Siegel D. Functions of NQO1 in cellular protection and CoQ(10) metabolism and its potential role as a redox sensitive molecular switch. Front Physiol. (2017) 8:595. 10.3389/fphys.2017.00595 28883796 PMC5573868

[181] Pittenger MF Mackay AM Beck SC Jaiswal RK Douglas R Mosca JD. Multilineage potential of adult human mesenchymal stem cells. Science. (1999) 284:143–7. 10.1126/science.284.5411.143 10102814

[182] Wada N Gronthos S Bartold PM. Immunomodulatory effects of stem cells. Periodontol. (2013) 63:198–216. 10.1111/prd.12024 23931061

[183] Nguyen TM Arthur A Hayball JD Gronthos S. EphB and Ephrin-B interactions mediate human mesenchymal stem cell suppression of activated T-cells. Stem Cells Dev. (2013) 22:2751–64. 10.1089/scd.2012.0676 23711177 PMC3787464

[184] Badiavas EV Abedi M Butmarc J Falanga V Quesenberry P. Participation of bone marrow derived cells in cutaneous wound healing. J Cell Physiol. (2003) 196:245–50. 10.1002/jcp.10260 12811816

[185] Awad HA Butler DL Boivin GP Smith FN Malaviya P Huibregtse B. Autologous mesenchymal stem cell-mediated repair of tendon. Tissue Eng. (1999) 5:267–77. 10.1089/ten.1999.5.267 10434073

[186] Squillaro T Peluso G Galderisi U. Clinical trials with mesenchymal stem cells: an update. Cell Transplant. (2016) 25:829–48. 10.3727/096368915X689622 26423725

[187] Pittenger MF Discher DE Péault BM Phinney DG Hare JM Caplan AI. Mesenchymal stem cell perspective: cell biology to clinical progress. NPJ Regen Med. (2019) 4:22. 10.1038/s41536-019-0083-6 31815001 PMC6889290

[188] Musiał-Wysocka A Kot M Majka M. The pros and cons of mesenchymal stem cell-based therapies. Cell Transplant. (2019) 28:801–12. 10.1177/0963689719837897 31018669 PMC6719501

[189] Yin K Wang S Zhao RC. Exosomes from mesenchymal stem/stromal cells: a new therapeutic paradigm. Biomark Res. (2019) 7:8. 10.1186/s40364-019-0159-x 30992990 PMC6450000

[190] El-Derany MO AbdelHamid SG. Upregulation of miR-96-5p by bone marrow mesenchymal stem cells and their exosomes alleviate non-alcoholic steatohepatitis: Emphasis on caspase-2 signaling inhibition. Biochem Pharmacol. (2021) 190:114624. 10.1016/j.bcp.2021.114624 34052187

[191] Davis C Dukes A Drewry M Helwa I Johnson MH Isales CM. MicroRNA-183-5p increases with age in bone-derived extracellular vesicles, suppresses bone marrow stromal (stem) cell proliferation, and induces stem cell senescence. Tissue Eng A. (2017) 23:1231–40. 10.1089/ten.tea.2016.0525 PMC5689127 28363268

[192] Desgagné V Bouchard L Guérin R. microRNAs in lipoprotein and lipid metabolism: from biological function to clinical application. Clin Chem Lab Med. (2017) 55:667–86. 10.1515/cclm-2016-0575 27987357

[193] Green CD Huang Y Dou X Yang L Liu Y Han JJ. Impact of dietary interventions on noncoding RNA networks and mRNAs encoding chromatin-related factors. Cell Rep. (2017) 18:2957–68. 10.1016/j.celrep.2017.03.001 28329687

[194] Machado MV Michelotti GA Pereira Tde A Boursier J Kruger L Swiderska-Syn M. Reduced lipoapoptosis, hedgehog pathway activation and fibrosis in caspase-2 deficient mice with non-alcoholic steatohepatitis. Gut. (2015) 64:1148–57. 10.1136/gutjnl-2014-307362 25053716 PMC4303564

[195] El-Derany MO El-Demerdash E. Pyrvinium pamoate attenuates non-alcoholic steatohepatitis: Insight on hedgehog/Gli and Wnt/β-catenin signaling crosstalk. Biochem Pharmacol. (2020) 177:113942. 10.1016/j.bcp.2020.113942 32240652

[196] Seki E Schwabe RF. Hepatic inflammation and fibrosis: functional links and key pathways. Hepatology. (2015) 61:1066–79. 10.1002/hep.27332 25066777 PMC4306641

[197] Tacke F Zimmermann HW. Macrophage heterogeneity in liver injury and fibrosis. J Hepatol. (2014) 60:1090–6. 10.1016/j.jhep.2013.12.025 24412603

[198] Ismail N Wang Y Dakhlallah D Moldovan L Agarwal K Batte K. Macrophage microvesicles induce macrophage differentiation and miR-223 transfer. Blood. (2013) 121:984–95. 10.1182/blood-2011-08-374793 23144169 PMC3567345

[199] Li M He Y Zhou Z Ramirez T Gao Y Gao Y. MicroRNA-223 ameliorates alcoholic liver injury by inhibiting the IL-6-p47 (phox)-oxidative stress pathway in neutrophils. Gut. (2017) 66:705–15. 10.1136/gutjnl-2016-311861 27679493 PMC5458746

[200] Neudecker V Brodsky KS Clambey ET Schmidt EP Packard TA Davenport B. Neutrophil transfer of miR-223 to lung epithelial cells dampens acute lung injury in mice. Sci Transl Med. (2017) 9:aah5360. 10.1126/scitranslmed.aah5360 PMC5842431 28931657

[201] Isola AL Chen S. Exosomes: the messengers of health and disease. Curr Neuropharmacol. (2017) 15:157–65. 10.2174/1570159X14666160825160421 27568544 PMC5327461

[202] Abenavoli L Peta V. Role of adipokines and cytokines in non-alcoholic fatty liver disease. Rev Recent Clin Trials. (2014) 9:134–40. 10.2174/1574887109666141216102458 25514909

[203] Stojsavljević S Gomerčić Palčić M Virović Jukić L Smirčić Duvnjak L Duvnjak M. Adipokines and proinflammatory cytokines, the key mediators in the pathogenesis of nonalcoholic fatty liver disease. World J Gastroenterol. (2014) 20:18070–91. 10.3748/wjg.v20.i48.18070 25561778 PMC4277948

[204] Azzu V Vacca M Virtue S Allison M Vidal-Puig A. Adipose tissue-liver cross talk in the control of whole-body metabolism: implications in nonalcoholic fatty liver disease. Gastroenterology. (2020) 158:1899–912. 10.1053/j.gastro.2019.12.054 32061598

[205] Wang S Luan J Lv X. Inhibition of endoplasmic reticulum stress attenuated ethanol-induced exosomal miR-122 and acute liver injury in mice. Alcohol Alcohol. (2019) 54:465–71. 10.1093/alcalc/agz058 31361816

[206] Fabregat I Moreno-Càceres J Sánchez A Dooley S Dewidar B Giannelli G. TGF-β signalling and liver disease. FEBS J. (2016) 283:2219–32. 10.1111/febs.13665 26807763

[207] Yang L Roh YS Song J Zhang B Liu C Loomba R. Transforming growth factor beta signaling in hepatocytes participates in steatohepatitis through regulation of cell death and lipid metabolism in mice. Hepatology. (2014) 59:483–95. 10.1002/hep.26698 23996730 PMC3946696

[208] Ottaviani S Stebbing J Frampton AE Zagorac S Krell J de Giorgio A. TGF-β induces miR-100 and miR-125b but blocks let-7a through LIN28B controlling PDAC progression. Nat Commun. (2018) 9:1845. 10.1038/s41467-018-03962-x 29748571 PMC5945639

[209] McCommis KS Hodges WT Brunt EM Nalbantoglu I McDonald WG Holley C. Targeting the mitochondrial pyruvate carrier attenuates fibrosis in a mouse model of nonalcoholic steatohepatitis. Hepatology. (2017) 65:1543–56. 10.1002/hep.29025 28027586 PMC5397348

[210] Park SW. Intestinal and hepatic niemann-pick c1-like 1. Diabetes Metab J. (2013) 37:240–8. 10.4093/dmj.2013.37.4.240 23991401 PMC3753488

[211] van Heek M Farley C Compton DS Hoos L Davis HR. Ezetimibe selectively inhibits intestinal cholesterol absorption in rodents in the presence and absence of exocrine pancreatic function. Br J Pharmacol. (2001) 134:409–17. 10.1038/sj.bjp.0704260 11564660 PMC1572957

[212] Sudhop T von Bergmann K. Cholesterol absorption inhibitors for the treatment of hypercholesterolaemia. Drugs. (2002) 62:2333–47. 10.2165/00003495-200262160-00002 12396226

[213] Park H Shima T Yamaguchi K Mitsuyoshi H Minami M Yasui K. Efficacy of long-term ezetimibe therapy in patients with nonalcoholic fatty liver disease. J Gastroenterol. (2011) 46:101–7. 10.1007/s00535-010-0291-8 20658156

[214] Yoneda M Fujita K Nozaki Y Endo H Takahashi H Hosono K. Efficacy of ezetimibe for the treatment of non-alcoholic steatohepatitis: An open-label, pilot study. Hepatol Res. (2010) 40:566–73. 10.1111/j.1872-034X.2010.00644.x 20412324

[215] Yamamura T Ohsaki Y Suzuki M Shinohara Y Tatematsu T Cheng J. Inhibition of Niemann-Pick-type C1-like1 by ezetimibe activates autophagy in human hepatocytes and reduces mutant α1-antitrypsin Z deposition. Hepatology. (2014) 59:1591–9. 10.1002/hep.26930 24214142

[216] Kim SH Kim G Han DH Lee M Kim I Kim B. Ezetimibe ameliorates steatohepatitis via AMP activated protein kinase-TFEB-mediated activation of autophagy and NLRP3 inflammasome inhibition. Autophagy. (2017) 13:1767–81. 10.1080/15548627.2017.1356977 28933629 PMC5640190

[217] Luan X Sansanaphongpricha K Myers I Chen H Yuan H Sun D. Engineering exosomes as refined biological nanoplatforms for drug delivery. Acta Pharmacol Sin. (2017) 38:754–63. 10.1038/aps.2017.12 28392567 PMC5520184

[218] Vader P Mol EA Pasterkamp G Schiffelers RM. Extracellular vesicles for drug delivery. Adv Drug Deliv Rev. (2016) 106:148–56. 10.1016/j.addr.2016.02.006 26928656

[219] Malhotra H Sheokand N Kumar S Chauhan AS Kumar M Jakhar P. Exosomes: tunable nano vehicles for macromolecular delivery of transferrin and lactoferrin to specific intracellular compartment. J Biomed Nanotechnol. (2016) 12:1101–14. 10.1166/jbn.2016.2229 27305829

[220] Wiklander OP Nordin JZ O'Loughlin A Gustafsson Y Corso G Mäger I. Extracellular vesicle *in vivo* biodistribution is determined by cell source, route of administration and targeting. J Extracell Vesicles. (2015) 4:26316. 10.3402/jev.v4.26316 25899407 PMC4405624

[221] Hood JL. Post isolation modification of exosomes for nanomedicine applications. Nanomedicine. (2016) 11:1745–56. 10.2217/nnm-2016-0102 27348448 PMC4941124

[222] Goh WJ Zou S Ong WY Torta F Alexandra AF Schiffelers RM. Bioinspired cell-derived nanovesicles versus exosomes as drug delivery systems: a cost-effective alternative. Sci Rep. (2017) 7:14322. 10.1038/s41598-017-14725-x 29085024 PMC5662560

[223] Marcus ME Leonard JN. FedExosomes: engineering therapeutic biological nanoparticles that truly deliver. Pharmaceuticals. (2013) 6:659–80. 10.3390/ph6050659 23894228 PMC3722064

[224] Zhang L Zhang S Yao J Lowery FJ Zhang Q Huang WC. Microenvironment-induced PTEN loss by exosomal microRNA primes brain metastasis outgrowth. Nature. (2015) 527:100–4. 10.1038/nature15376 26479035 PMC4819404

[225] Deng ZB Poliakov A Hardy RW Clements R Liu C Liu Y. Adipose tissue exosome-like vesicles mediate activation of macrophage-induced insulin resistance. Diabetes. (2009) 58:2498–505. 10.2337/db09-0216 19675137 PMC2768161

[226] Xue J Yang J Luo M Cho WC Liu X. MicroRNA-targeted therapeutics for lung cancer treatment. Expert Opin Drug Discov. (2017) 12:141–57. 10.1080/17460441.2017.1263298 27866431

[227] Pretti MAM Bernardes SS da Cruz JGV Boroni M Possik PA. Extracellular vesicle-mediated crosstalk between melanoma and the immune system: Impact on tumor progression and therapy response. J Leukoc Biol. (2020) 108:1101–15. 10.1002/JLB.3MR0320-644R 32450618

[228] Jin JO Kim G Hwang J Han KH Kwak M Lee PCW. Nucleic acid nanotechnology for cancer treatment. Biochim Biophys Acta Rev Cancer. (2020) 1874:188377. 10.1016/j.bbcan.2020.188377 32418899

[229] van Zandwijk N Pavlakis N Kao SC Linton A Boyer MJ Clarke S. Safety and activity of microRNA-loaded minicells in patients with recurrent malignant pleural mesothelioma: a first-in-man, phase 1, open-label, dose-escalation study. Lancet Oncol. (2017) 18:1386–96. 10.1016/S1470-2045(17)30621-6 28870611

[230] Yang C Zhang M Merlin D. Advances in plant-derived edible nanoparticle-based lipid nano-drug delivery systems as therapeutic nanomedicines. J Mater Chem B. (2018) 6:1312–21. 10.1039/C7TB03207B 30034807 PMC6053076

[231] Zhang M Viennois E Xu C Merlin D. Plant derived edible nanoparticles as a new therapeutic approach against diseases. Tissue Barriers. (2016) 4:e1134415. 10.1080/21688370.2015.1134415 27358751 PMC4910829

[232] Ju S Mu J Dokland T Zhuang X Wang Q Jiang H. Grape exosome-like nanoparticles induce intestinal stem cells and protect mice from DSS-induced colitis. Mol Ther. (2013) 21:1345–57. 10.1038/mt.2013.64 23752315 PMC3702113

[233] Wang B Zhuang X Deng ZB Jiang H Mu J Wang Q. Targeted drug delivery to intestinal macrophages by bioactive nanovesicles released from grapefruit. Mol Ther. (2014) 22:522–34. 10.1038/mt.2013.190 23939022 PMC3944329

[234] Wang Q Ren Y Mu J Egilmez NK Zhuang X Deng Z. Grapefruit-derived nanovectors use an activated leukocyte trafficking pathway to deliver therapeutic agents to inflammatory tumor sites. Cancer Res. (2015) 75:2520–9. 10.1158/0008-5472.CAN-14-3095 25883092 PMC4470740

[235] Brahmbhatt M Gundala SR Asif G Shamsi SA Aneja R. Ginger phytochemicals exhibit synergy to inhibit prostate cancer cell proliferation. Nutr Cancer. (2013) 65:263–72. 10.1080/01635581.2013.749925 23441614 PMC3925258

[236] Raimondo S Naselli F Fontana S Monteleone F Lo Dico A Saieva L. Citrus limon-derived nanovesicles inhibit cancer cell proliferation and suppress CML xenograft growth by inducing TRAIL-mediated cell death. Oncotarget. (2015) 6:19514–27. 10.18632/oncotarget.4004 26098775 PMC4637302

[237] Deng Z Rong Y Teng Y Mu J Zhuang X Tseng M. Broccoli-derived nanoparticle inhibits mouse colitis by activating dendritic cell AMP-activated protein kinase. Mol Ther. (2017) 25:1641–54. 10.1016/j.ymthe.2017.01.025 28274798 PMC5498816

[238] Mu J Zhuang X Wang Q Jiang H Deng ZB Wang B. Interspecies communication between plant and mouse gut host cells through edible plant derived exosome-like nanoparticles. Mol Nutr Food Res. (2014) 58:1561–73. 10.1002/mnfr.201300729 24842810 PMC4851829

[239] Zhao Z Yu S Li M Gui X Li P. Isolation of exosome-like nanoparticles and analysis of MicroRNAs derived from coconut water based on small RNA high-throughput sequencing. J Agric Food Chem. (2018) 66:2749–57. 10.1021/acs.jafc.7b05614 29478310

[240] Yu S Zhao Z Xu X Li M Li P. Characterization of three different types of extracellular vesicles and their impact on bacterial growth. Food Chem. (2019) 272:372–8. 10.1016/j.foodchem.2018.08.059 30309557

[241] Fujita D Arai T Komori H Shirasaki Y Wakayama T Nakanishi T. Apple-derived nanoparticles modulate expression of organic-anion-transporting polypeptide (OATP) 2B1 in Caco-2 cells. Mol Pharm. (2018) 15:5772–80. 10.1021/acs.molpharmaceut.8b00921 30359033

[242] An Q Hückelhoven R Kogel KH van Bel AJ. Multivesicular bodies participate in a cell wall-associated defence response in barley leaves attacked by the pathogenic powdery mildew fungus. Cell Microbiol. (2006) 8:1009–19. 10.1111/j.1462-5822.2006.00683.x 16681841

[243] An Q van Bel AJ Hückelhoven R. Do plant cells secrete exosomes derived from multivesicular bodies? Plant Signal Behav. (2007) 2:4–7. 10.4161/psb.2.1.3596 19704795 PMC2633885

[244] Zhang M Viennois E Prasad M Zhang Y Wang L Zhang Z. Edible ginger-derived nanoparticles: A novel therapeutic approach for the prevention and treatment of inflammatory bowel disease and colitis-associated cancer. Biomaterials. (2016) 101:321–40. 10.1016/j.biomaterials.2016.06.018 27318094 PMC4921206

[245] Zhang M Xiao B Wang H Han MK Zhang Z Viennois E. Edible ginger-derived nano-lipids loaded with doxorubicin as a novel drug-delivery approach for colon cancer therapy. Mol Ther. (2016) 24:1783–96. 10.1038/mt.2016.159 27491931 PMC5112046

[246] Zhuge Q Zhang Y Liu B Wu M. Blueberry polyphenols play a preventive effect on alcoholic fatty liver disease C57BL/6 J mice by promoting autophagy to accelerate lipolysis to eliminate excessive TG accumulation in hepatocytes. Ann Palliat Med. (2020) 9:1045–54. 10.21037/apm.2020.03.38 32389004

[247] Khalid S Barfoot KL May G Lamport DJ Reynolds SA Williams CM. Effects of acute blueberry flavonoids on mood in children and young adults. Nutrients. (2017) 9:20158. 10.3390/nu9020158 PMC5331589 28230732

[248] Shi M Loftus H McAinch AJ Su XQ. Blueberry as a source of bioactive compounds for the treatment of obesity, type 2 diabetes and chronic inflammation. J Funct Foods. (2017) 30:16–29. 10.1016/j.jff.2016.12.036

[249] Wu T Gao Y Guo X Zhang M Gong L. Blackberry and blueberry anthocyanin supplementation counteract high-fat-diet-induced obesity by alleviating oxidative stress and inflammation and accelerating energy expenditure. Oxid Med Cell Longev. (2018) 2018:4051232. 10.1155/2018/4051232 30057677 PMC6051031

[250] Xu N Meng H Liu T Feng Y Qi Y Zhang D. Blueberry phenolics reduce gastrointestinal infection of patients with cerebral venous thrombosis by improving depressant-induced autoimmune disorder via miR-155-mediated brain-derived neurotrophic factor. Front Pharmacol. (2017) 8:853. 10.3389/fphar.2017.00853 29230173 PMC5712003

[251] Zepeda A Aguayo LG Fuentealba J Figueroa C Acevedo A Salgado P. Blueberry extracts protect testis from hypobaric hypoxia induced oxidative stress in rats. Oxid Med Cell Longev. (2012) 2012:975870. 10.1155/2012/975870 23213351 PMC3503460

[252] Zhao WJ Bian YP Wang QH Yin F Yin L Zhang YL. Blueberry-derived exosomes-like nanoparticles ameliorate nonalcoholic fatty liver disease by attenuating mitochondrial oxidative stress. Acta Pharmacol Sin. (2022) 43:645–58. 10.1038/s41401-021-00681-w 33990765 PMC8888548

[253] Mortezaee K. Human hepatocellular carcinoma: protection by melatonin. J Cell Physiol. (2018) 233:6486–508. 10.1002/jcp.26586 29672851

[254] Xu P Wang J Hong F Wang S Jin X Xue T. Melatonin prevents obesity through modulation of gut microbiota in mice. J Pineal Res. (2017) 62:12399. 10.1111/jpi.12399 28199741

[255] Liu Z Gan L Xu Y Luo D Ren Q Wu S. Melatonin alleviates inflammasome-induced pyroptosis through inhibiting NF-κB/GSDMD signal in mice adipose tissue. J Pineal Res. (2017) 63:12414. 10.1111/jpi.12414 28398673

[256] Fernández Vázquez G Reiter RJ Agil A. Melatonin increases brown adipose tissue mass and function in Zücker diabetic fatty rats: implications for obesity control. J Pineal Res. (2018) 64:e12472. 10.1111/jpi.12472 29405372

[257] Rong B Feng R Liu C Wu Q Sun C. Reduced delivery of epididymal adipocyte-derived exosomal resistin is essential for melatonin ameliorating hepatic steatosis in mice. J Pineal Res. (2019) 66:e12561. 10.1111/jpi.12561 30659651

[258] Sun L Xu R Sun X Duan Y Han Y Zhao Y. Safety evaluation of exosomes derived from human umbilical cord mesenchymal stromal cell. Cytotherapy. (2016) 18:413–22. 10.1016/j.jcyt.2015.11.018 26857231

[259] Ding J Wang J Chen J. Exosomes as therapeutic vehicles in liver diseases. Ann Transl Med. (2021) 9:735. 10.21037/atm-20-5422 33987433 PMC8106083

[260] Pritchard CC Kroh E Wood B Arroyo JD Dougherty KJ Miyaji MM. Blood cell origin of circulating microRNAs: a cautionary note for cancer biomarker studies. Cancer Prev Res. (2012) 5:492–7. 10.1158/1940-6207.CAPR-11-0370 PMC4186243 22158052

[261] Wisgrill L Lamm C Hartmann J Preißing F Dragosits K Bee A. Peripheral blood microvesicles secretion is influenced by storage time, temperature, and anticoagulants. Cytometry A. (2016) 89:663–72. 10.1002/cyto.a.22892 27442840

[262] Li YJ Wu JY Liu J Xu W Qiu X Huang S. Artificial exosomes for translational nanomedicine. J Nanobiotechnology. (2021) 19:242. 10.1186/s12951-021-00986-2 34384440 PMC8359033

